# Modulation of DBS-induced cortical responses and movement by the directionality and magnitude of current administered

**DOI:** 10.1038/s41531-024-00663-9

**Published:** 2024-03-08

**Authors:** Rachel K. Spooner, Baccara J. Hizli, Bahne H. Bahners, Alfons Schnitzler, Esther Florin

**Affiliations:** 1 Institute of Clinical Neuroscience and Medical Psychology, Medical Faculty and University Hospital Düsseldorf, Heinrich Heine University, Düsseldorf, Germany; 2 Department of Neurology, Center for Movement Disorders and Neuromodulation, Medical Faculty and University Hospital Düsseldorf, Heinrich Heine University, Düsseldorf, Germany

**Keywords:** Parkinson's disease, Basal ganglia, Neurophysiology

## Abstract

Subthalamic deep brain stimulation (STN-DBS) is an effective therapy for alleviating motor symptoms in people with Parkinson’s disease (PwP), although some may not receive optimal clinical benefits. One potential mechanism of STN-DBS involves antidromic activation of the hyperdirect pathway (HDP), thus suppressing cortical beta synchrony to improve motor function, albeit the precise mechanisms underlying optimal DBS parameters are not well understood. To address this, 18 PwP with STN-DBS completed a 2 Hz monopolar stimulation of the left STN during MEG. MEG data were imaged in the time-frequency domain using minimum norm estimation. Peak vertex time series data were extracted to interrogate the directional specificity and magnitude of DBS current on evoked and induced cortical responses and accelerometer metrics of finger tapping using linear mixed-effects models and mediation analyses. We observed increases in evoked responses (HDP ~ 3–10 ms) and synchronization of beta oscillatory power (14–30 Hz, 10–100 ms) following DBS pulse onset in the primary sensorimotor cortex (SM1), supplementary motor area (SMA) and middle frontal gyrus (MFG) ipsilateral to the site of stimulation. DBS parameters significantly modulated neural and behavioral outcomes, with clinically effective contacts eliciting significant increases in medium-latency evoked responses, reductions in induced SM1 beta power, and better movement profiles compared to suboptimal contacts, often regardless of the magnitude of current applied. Finally, HDP-related improvements in motor function were mediated by the degree of SM1 beta suppression in a setting-dependent manner. Together, these data suggest that DBS-evoked brain-behavior dynamics are influenced by the level of beta power in key hubs of the basal ganglia-cortical loop, and this effect is exacerbated by the clinical efficacy of DBS parameters. Such data provides novel mechanistic and clinical insight, which may prove useful for characterizing DBS programming strategies to optimize motor symptom improvement in the future.

## Introduction

While the advent of deep brain stimulation of the subthalamic nucleus (STN-DBS) has proven highly effective for temporarily alleviating motor symptoms in people with Parkinson’s disease (PwP), therapeutic outcomes vary widely across individuals^1^, in part due to the large parameter space required to be individually titrated to optimize clinical outcomes. For example, parameters such as the directionality or magnitude of current administered throughout the electrode may help augment the efficacy of DBS programming strategies by focusing and emphasizing current spread to the appropriate subcortical entities^2–6^, albeit clinicians currently lack reliable biomarkers for indexing effective parameter settings in movement disorder patients.

In regard to the brain, one proposed mechanism underlying clinical outcomes induced by STN-DBS is an antidromic activation of the hyperdirect pathway (HDP), which subsequently suppresses pathologically elevated beta (~15–30 Hz) synchrony in key hubs of the basal ganglia–cortical loop to improve motor function in model systems^7–13^, albeit the direct effect of DBS parameter settings on this pathway has yet to be comprehensively understood in humans. To date, human studies of STN-DBS using electrocorticography (ECoG) or magneto-/electro-encephalography (M/EEG) have identified the presence of medium-latency (~2–10 ms, recent review of DBS-evoked potential latencies and their relation to HDP activation^14^) evoked cortical responses likely reflective of HDP activation based on its conduction speed and topography which localize to a distributed sensorimotor-prefrontal network including primary motor (M1) and somatosensory (S1) cortices, supplementary motor areas (SMA) and prefrontal regions^2,3,8,9,14–16^. Moreover, the lack of medium-latency potentials evoked by alternative DBS strategies for PwP (e.g., pallidal DBS) supports the notion of medium-latency cortical responses underlying STN-related activation of the HDP to induce clinical outcomes^8^. Similarly, electrophysiological evidence using invasive and non-invasive recording techniques demonstrates the presence of pathologically elevated beta oscillations, as well as elevated STN-cortical coherence in the beta frequency range, which can be suppressed to augment motor performance during high-frequency STN-DBS paradigms^17–22^. However, the comprehensive impact of these neurophysiological features for indexing DBS programming efficacy and motor outcomes in PwP has yet to be evaluated.

To this end, we enrolled PwP with STN-DBS to comprehensively quantify proposed neural and behavioral correlates of clinical outcomes relating to the clinical efficacy of DBS parameters, including the directionality (i.e., contact) and magnitude (i.e., stimulation amplitude) of current administration. Briefly, prior work by our lab and others has demonstrated a differential impact of DBS parameters, including optimal contact orientations and larger stimulation amplitudes on aforementioned neurophysiological markers, which relate to improved motor performance or, alternatively, DBS-induced side effects in PwP^2,3,8^. However, this study expands upon prior relevant work by evaluating effective parameter spaces on DBS-evoked and induced (i.e., oscillatory) activity in concert, rather than in isolation, to provide a comprehensive mechanistic and clinical insight into STN-DBS for PwP. Specifically, PwP completed a low-frequency monopolar stimulation paradigm of the left STN during MEG to directly quantify neural dynamics evoked and induced by STN-DBS as a function of varying parameter settings (i.e., best and worst contact settings, increasing stimulation amplitudes) using linear mixed-effects models (LME). Finally, using mediation analyses, we probed a well-theorized mechanism of action of STN-DBS (i.e., HDP-related improvements in motor function through levels of cortical beta synchrony) using quantitative, accelerometer-based recordings of standardized movements (i.e., Movement Disorder Society Unified Parkinson’s Disease Rating Scale III: MDS-UPDRS III Item 3.4). We hypothesized that better DBS parameter settings (e.g., clinically effective contacts and stimulation amplitudes) would elicit larger medium-latency sensorimotor evoked responses, reductions in cortical beta synchrony, and improved finger tapping performance compared to less optimal parameter settings. Moreover, we hypothesized that HDP-related improvements in motor function (i.e., DBS-evoked brain-behavior interactions) would be mediated by the level of beta power in the sensorimotor system, which would be differentially impacted based on the clinical efficacy of current administered to the STN.

## Results

Of the 20 PwP enrolled in the current study, 2 participants were unable to successfully complete the MEG aspects of the study. The remaining 18 PwP had a mean age of 62.6 years (3 females).

### Sensor-level analysis

**Fig. 1:**
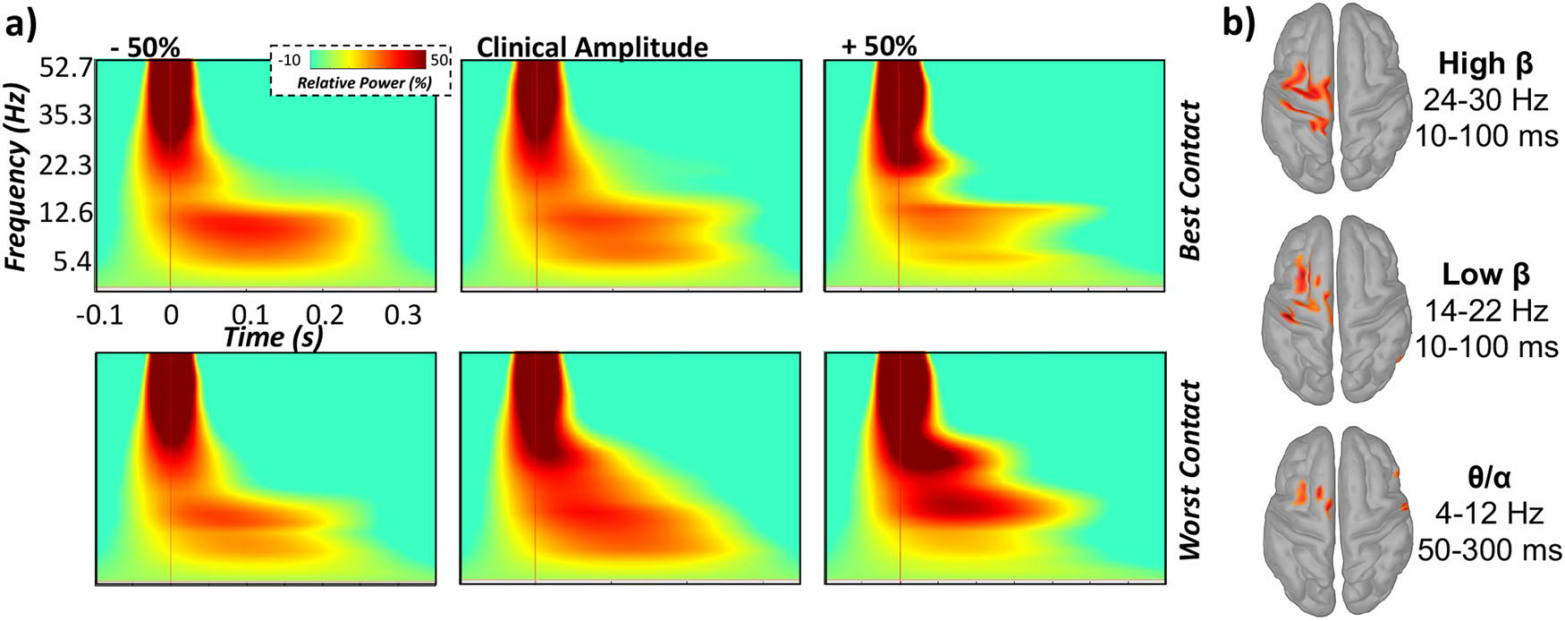
Neural responses induced by STN-DBS during different DBS parameter settings. **a** Grand-averaged time-frequency spectrograms over the sensorimotor cortex ipsilateral to the site of STN stimulation (MEG0432). DBS-induced cortical responses are displayed for best and worst DBS contacts (top and bottom panels, respectively), which were tested at clinically effective stimulation amplitudes (±50% clinical stimulation amplitude). Time-point zero denotes DBS pulse onset, while the baseline was defined as the −100 to −5 ms window prior to stimulation onset. Of note, oscillatory response time windows were shifted by at least 10 ms surrounding response maxima (i.e., greatest amplitude change from baseline) following DBS pulse onset for subsequent source analyses to avoid remnants of the DBS artifact and to optimize the signal-to-noise ratio. **b** Grand-averaged cortical reconstructions of theta-alpha (4–12 Hz), low beta (14–22 Hz), and high beta (24–30 Hz) induced cortical responses from ~10 to 300 ms following DBS pulse onset. For more information regarding significant time-frequency windows identified from grand-averaged sensor level data, see (Supplementary Fig. 1).

Time-frequency analyses indicated significant DBS-induced oscillatory responses in the theta-alpha (4–12 Hz), low beta (14–22 Hz), and high beta (24–30 Hz) ranges. These responses were robust in gradiometers near the ipsilateral sensorimotor strip across all participants and experimental sessions (nonparametric permutation *t*-test*: p*_corrected_ < 0.005; Fig. 1). Specifically, sustained synchronizations in theta-alpha activity were observed during the 50–300 ms time window following DBS pulse onset. In contrast, transient increases in low and high beta oscillatory activity (i.e., 14–22 and 24–30 Hz, respectively) were detected during the 100 ms time window immediately following DBS pulse onset. Importantly, to assess the contribution of the evoked, phase-locked signal, we re-ran the sensor-level analyses with the time-domain averaged signal regressed out. This analysis indicated almost identical time–frequency windows, suggesting that all three DBS-induced cortical responses comprised predominantly non-phase-locked oscillatory activity.

In regard to the time-domain analyses, significant medium-latency evoked neural responses were found in many sensors near the ipsilateral sensorimotor regions from 3 to 7 ms following DBS pulse onset (nonparametric permutation *t*-test*: p*_corrected_ < 0.005; Fig. 4). Of note, due to remnants of the DBS artifact, we did not evaluate evoked nor induced cortical response dynamics at latencies shorter than 2 ms.

### STN-DBS parameter settings modulate evoked and oscillatory profiles in the sensorimotor network

To identify the neural origins of oscillations seen at the sensor level, these windows were imaged and transformed in the time-frequency domain using MNE and Morlet wavelets. The resulting maps indicated that all three oscillatory responses reliably localized to a distributed sensorimotor network ipsilateral to the site of stimulation, including the primary motor (M1) and somatosensory (S1) cortices, supplementary motor area (SMA) and middle frontal gyrus (MFG), regardless of experimental session (Fig. 1). As described in the methods, we next extracted peak oscillatory activity from each respective grand-averaged cluster (i.e., across all participants, experimental sessions and trials) in the left M1, S1, SMA, and MFG for each experimental session separately to examine the influence of DBS parameter settings (i.e., directionality and magnitude of DBS current) on induced neural activity using linear mixed-effects models (LME).

**Fig. 2:**
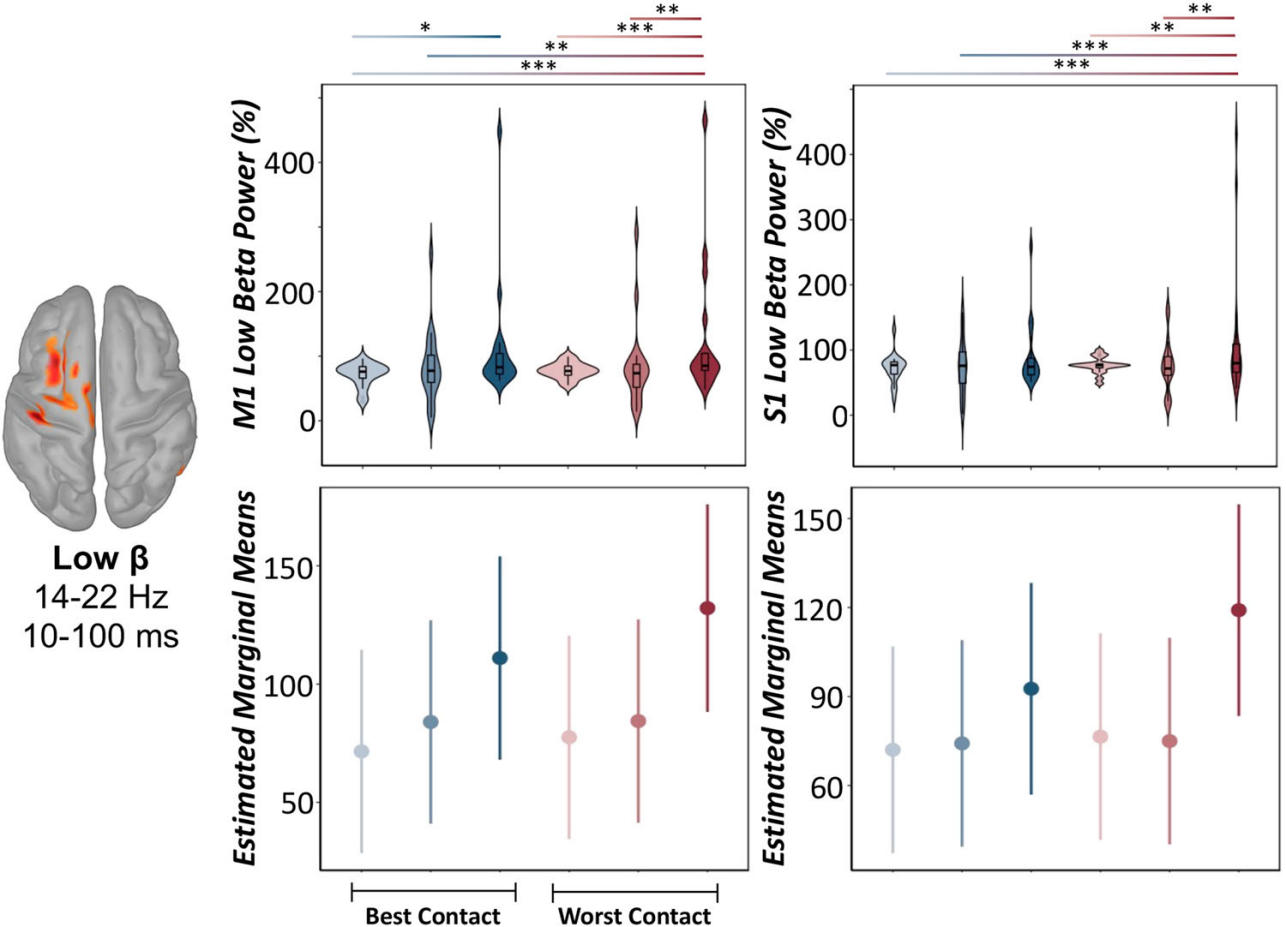
Modulation of DBS-induced low beta cortical responses by varying DBS parameters. (Top): Peak vertex time series data extracted from the ipsilateral primary motor (M1: left panel) and somatosensory (S1: right panel) cortices revealed significant modulation by varying DBS parameter settings in the low beta frequency range (14–22 Hz). Color gradient from light to dark indicates −50%, clinically effective, and +50% amplitude definitions, respectively. Violin plots include a combined box plot (box edges: first 25th percentile quartile to third 75th percentile quartile; center line: median; data minima/maxima: whisker length) and histogram distribution of each raw oscillatory response. (Bottom): Estimated marginal means and 95% confidence intervals of M1 (left) and S1 (right) low beta oscillatory power from linear mixed-effects models of each low beta oscillatory response as a function of the experimental session. Post hoc significance is fixed for top and bottom panels, and color gradients from light to dark and blue to red denote significant post hoc effects across each experimental session. LME: *pcorrected < 0.05, **pcorrected < 0.01, ***pcorrected < .005. For more details regarding post hoc analyses, see (Supplementary Tables 5 and 6).

**Fig. 3:**
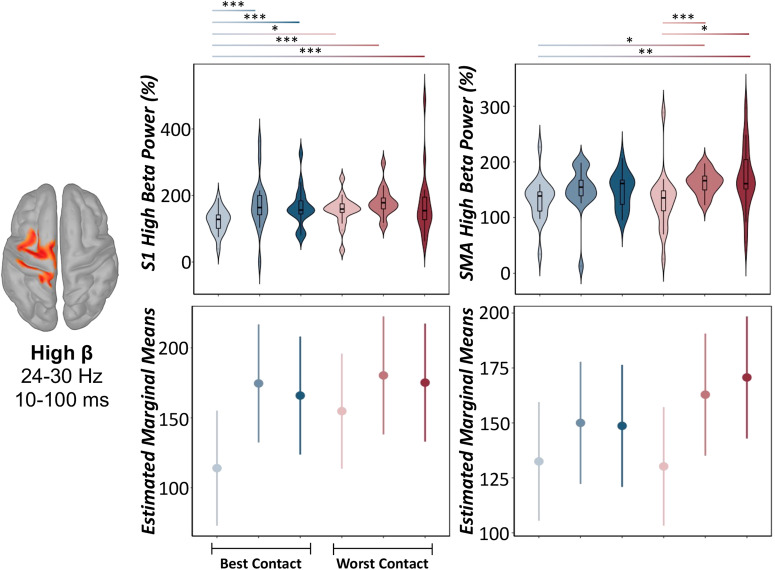
Modulation of DBS-induced high beta cortical responses by varying DBS parameters. (Top): Peak vertex time series data extracted from the ipsilateral primary somatosensory (S1: left panel) cortex and supplementary motor area (SMA: right panel) revealed significant modulation by varying DBS parameter settings in the high beta frequency range (24–30 Hz). Color gradient from light to dark indicates −50%, clinically effective, and +50% amplitude definitions, respectively. Violin plots include a combined box plot (box edges: first 25th percentile quartile to third 75th percentile quartile; center line: median; data minima/maxima: whisker length) and histogram distribution of each oscillatory response. (Bottom): Estimated marginal means and 95% confidence intervals of S1 (left) and SMA (right) high beta oscillatory power from linear mixed-effects models of each low beta oscillatory response as a function of the experimental session. Post hoc significance is fixed for top and bottom panels, and color gradients from light to dark and blue to red denote significant post hoc effects across each experimental session. LME: *pcorrected < 0.05, **pcorrected < .01, ***pcorrected < 0.005. For more details regarding post hoc analyses, see (Supplementary Tables 10 and 12).

While DBS-induced increases in the theta-alpha range were unaffected by DBS parameter settings (LME: *ps*_corrected_ > 0.160; see Supplementary Tables 1–4), low and high beta oscillatory responses were ubiquitously modulated by DBS parameters in key areas of the ipsilateral sensorimotor network (i.e., M1, S1, and SMA). Specifically, we observed a main effect of experimental session on low beta oscillatory power in the left M1 (LME: *F*(88.2) = 3.41, *p* = 0.007, Cohen’s *d* = 0.39, 95% CI [0.03, 0.81]) and S1 (*F*(88.2) = 2.68, *p* = 0.026, Cohen’s *d* = 0.35, 95% CI [0.07, 0.77]), such that reductions in low beta oscillatory responses (i.e., less positive increases or weaker DBS-induced beta power, expressed as percent change from baseline) were observed during optimal contact settings compared to suboptimal ones, often regardless of stimulation amplitude (Fig. 2, Supplementary Tables 5–6). Additionally, we observed a main effect of DBS parameter settings on high beta power in the left S1 (LME: *F*(86.8) = 3.74, *p* = 0.004, Cohen’s d = 0.41, 95% CI [0.01, 0.84]) and SMA (LME: *F*(86.8) = 2.63, *p* = 0.029, Cohen’s *d* = 0.35, 95% CI [0.08, 0.77]). Generally, these results suggest that more effective DBS parameter settings, such as optimal contact settings and lower stimulation amplitudes, were associated with weaker oscillatory responses in the high beta band in a distributed ipsilateral sensorimotor network (Fig. 3, Supplementary Tables 10 and 12). Finally, the peak frequency of low and high-frequency beta synchrony in sensorimotor hubs was not significantly modulated by DBS parameters (LME: *p*s > 0.052, Supplementary Tables 13–20).

**Fig. 4:**
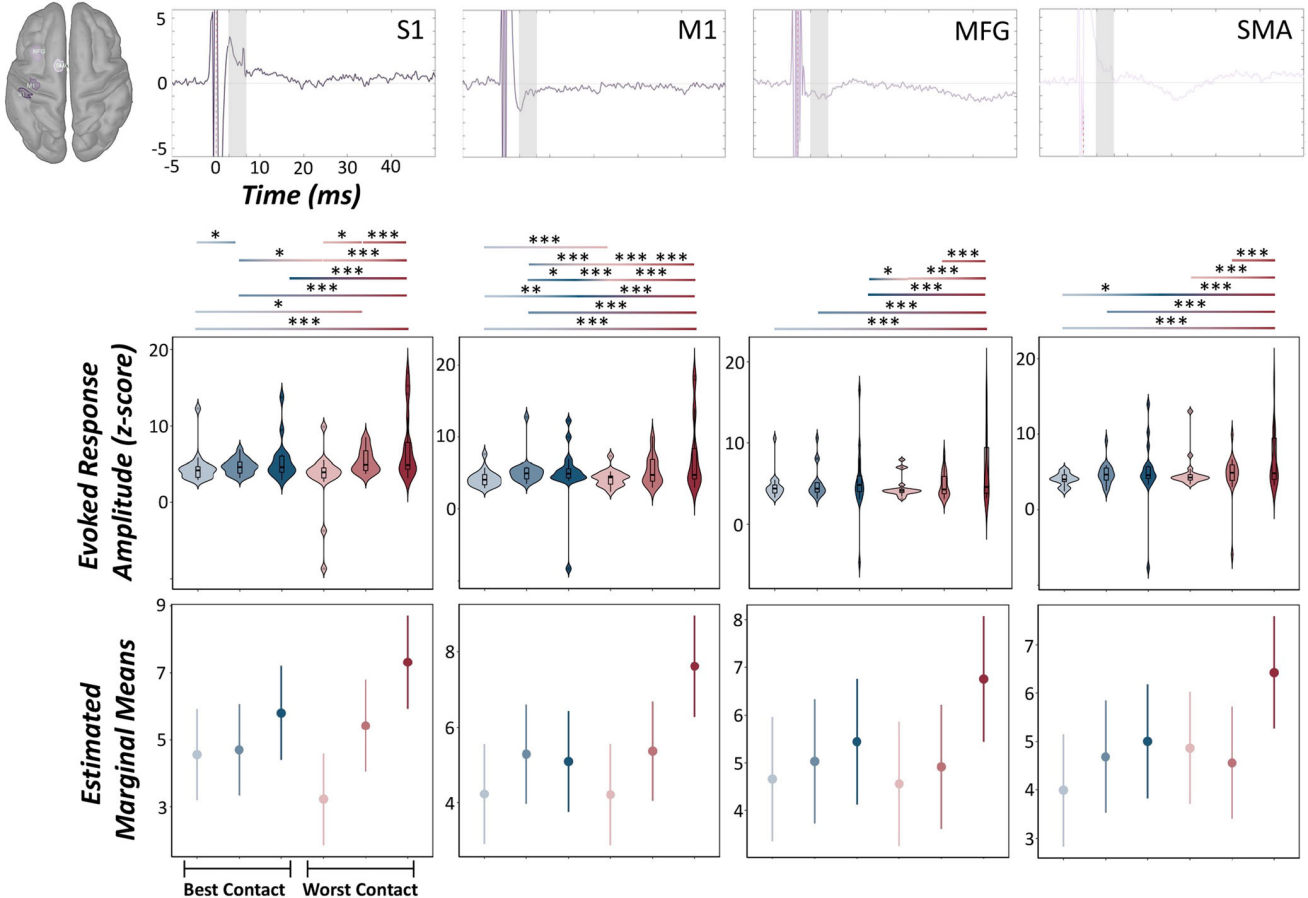
Medium-latency DBS-evoked cortical responses by varying DBS parameters. (Top): Peak vertex time series data extracted from the ipsilateral primary somatosensory (S1) cortex, primary motor (M1) cortex, middle frontal gyrus (MFG), and supplementary motor area (SMA) revealed significant modulation by varying DBS parameter settings during the 3–7 ms window following DBS pulse onset (gray shaded area; i.e., medium-latency evoked responses). (Middle): Color gradient from light to dark indicates −50%, clinically-effective and +50% amplitude definitions, respectively. Violin plots include a combined box plot (box edges: first 25th percentile quartile to third 75th percentile quartile; center line: median; data minima/maxima: whisker length), and histogram distribution of each evoked response. (Bottom): Estimated marginal means and 95% confidence intervals of S1, M1, MFG, and SMA medium-latency evoked responses from linear mixed-effects models of each evoked cortical response as a function of the experimental session. Post hoc significance is fixed for top and bottom panels, and color gradients from light to dark and blue to red denote significant post hoc effects across each experimental session. LME: *pcorrected < 0.05, **pcorrected < 0.01, ***pcorrected < 0.005. For more details regarding post hoc analyses, see (Supplementary Tables 21–24).

To interrogate the impact of STN-DBS parameter settings on DBS-evoked cortical responses, MNE source estimates were computed for medium-latency (3–7 ms) evoked responses observed at the sensor level for all participants and experimental sessions and subjected to LMEs. We observed significant main effects of the session on medium-latency evoked response amplitudes in M1, S1, MFG, and SMA (LME: *p’s* < 0.001, Cohen’s *d*’s > 0.27), such that sensorimotor evoked responses tended to scale linearly with increasing stimulation amplitude and were larger when undergoing clinically-effective contact settings compared to less effective ones (Fig. 4, Supplementary Tables 21–23).

### DBS-induced beta oscillations mediate HDP-related improvements in motor function in PwP

**Fig. 5:**
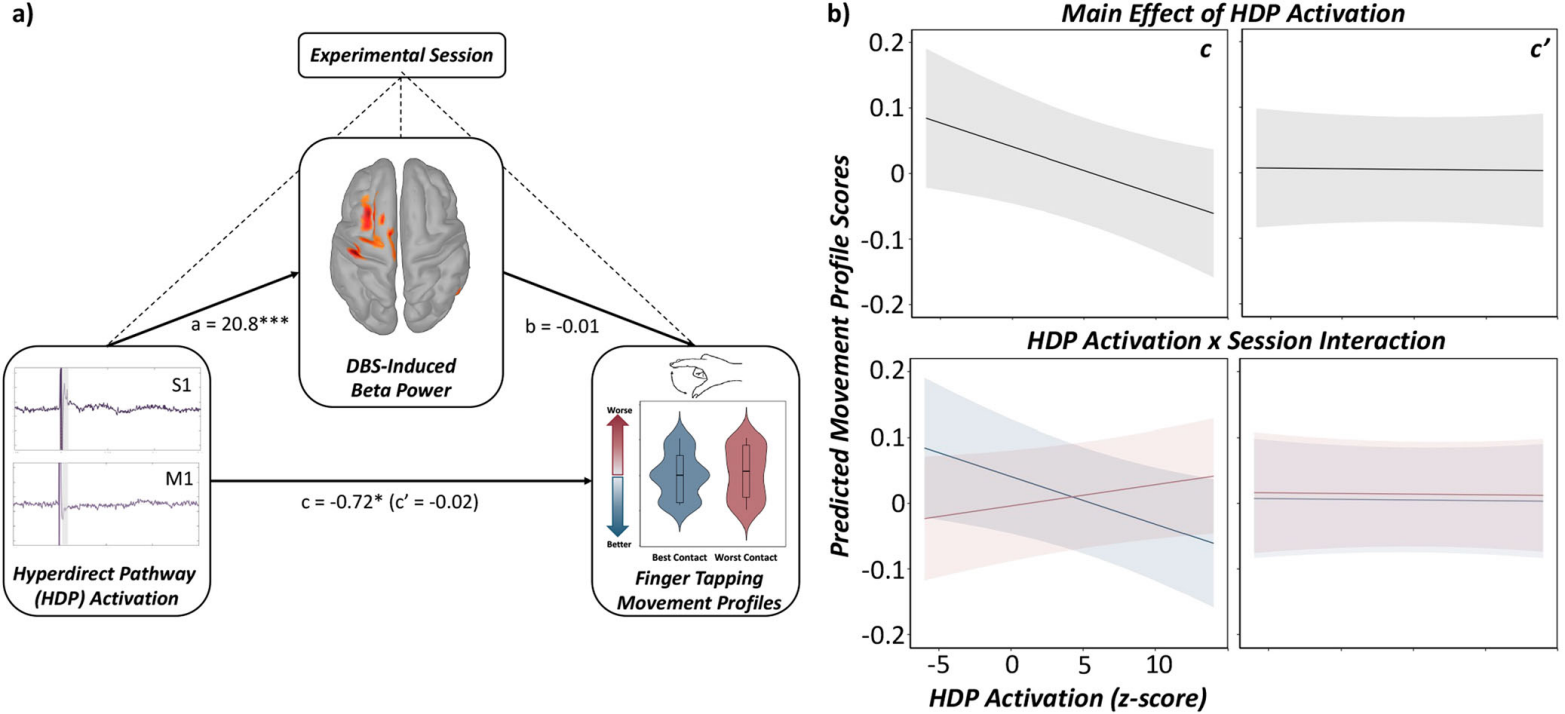
Sensorimotor cortical beta oscillations fully mediate HDP-related improvements in motor function. **a** Conceptual illustration denoting the statistical model probed in the current study to evaluate the mediation of quantitative motor outcomes by HDP-related cortical activations in sensorimotor cortices (i.e., primary motor and somatosensory cortices: SM1) through the mediator (i.e., SM1 beta power) while controlling for the experimental session (factor with 2 levels: best and worst contact at clinical amplitudes). Subject (factor with 11 levels) and ROI/frequency (factor with 3 levels: S1 low and high beta, M1 low beta) were included as a nested random effect. Unstandardized coefficients are displayed for each predictive path (a = independent variable (IV) to mediator, b = mediator to dependent variable (DV), c = direct effect of IV to DV, c’ = indirect effect of IV to DV through the mediator). Solid black arrows denote predictive paths, while dashed black lines denote modeling of covariates (i.e., experimental session on IV, mediator, and DV). (**b** top panel): The main effect of medium-latency SM1 evoked responses (i.e., HDP activation) on finger tapping movement profiles with (path c’) and without (path c) the mediator. (**b** bottom panel): Interaction effect of SM1 evoked responses and experimental session (i.e., blue indicates the best contact at clinically effective stimulation amplitudes, red indicates the worst directional contact at clinically effective stimulation amplitudes) on finger tapping movement profiles with (path c’) and without (path c) the mediator. A full mediation of HDP-related improvements in motor function through the mediator (i.e., DBS-induced beta power) was observed. Axes are fixed for each graph. LME: *p < 0.05, ***p < 0.001. For more details regarding post hoc analyses, see (Supplementary Table 26).

Next, we aimed to evaluate a well-theorized link between HDP-related evoked response amplitude, beta suppression, and motor outcomes in a subset of PwP who completed MDS-UPDRS finger tapping paradigms with an accelerometer affixed to the right index finger. As described in the methods and in our recent paper^2^, we constructed an empirical, sample-specific finger tapping movement profile score using an EFA of a compilation of accelerometer metrics pertinent to clinical outcomes of motor decline (i.e., acceleration magnitude, movement execution smoothness, movement pacing and the coefficient of variation in each metric). The initial EFA based on all six accelerometer metrics indicated a single-factor solution with moderate to good fit (*χ*^2^ = 1041.71, RMSEA = 0.40, 90% CI [0.38, 0.42], CFI = 0.69, SRMR = 0.15). Since tap frequency variability (i.e., coefficient of variation) loaded poorly onto the factor (*λ* = 0.34), excluding this variable yielded a single-factor solution with the superior model fit (*χ*^2^ = 614.00, RMSEA = 0.41, 90% CI [0.38, 0.44], CFI = 0.78, SRMR = 0.09). Thus, our empirically-derived, sample-specific quantification of finger-tapping movement profiles comprised of reverse coded acceleration magnitude, acceleration variability, reverse coded acceleration jerk (i.e., movement execution smoothness), movement smoothness variability, and tap frequency (i.e., inter-tap interval), which accounted for 70.0% of the variance in finger tapping movement profiles (for achieved loadings, see Supplementary Table 25). Of note, lower movement profile scores are reflective of smoother movements which was used as a dependent variable in our LME mediation analyses (Fig. 5).

Our results indicated a full mediation of the relationship between HDP-related improvements in motor function (i.e., larger medium-latency evoked response amplitude predictive of lower and better finger tapping movement profile scores) by the mediator (i.e., beta oscillatory power; Fig. 5, Supplementary Table 26), which suggests that the level of DBS-induced beta synchrony in primary somatosensory and motor cortices (SM1: i.e., S1 low and high beta and M1 low beta power) fully drives the observed HDP-related improvements in motor performance. Moreover, there was a differential impact on DBS-evoked brain-behavior relationships based on the experimental session, as evidenced by a significant evoked response x session interaction. Essentially, when stimulating the best directional contact at clinically effective amplitudes, we observed the hypothesized relationship between DBS-evoked cortical responses on behavior (i.e., larger evoked response amplitude related to better clinical outcomes), while the opposite trajectory was observed when stimulating at the worst directional contact at the same clinical amplitude. Finally, this relationship and its interaction with the experimental session, were effectively abolished when including the mediator (i.e., beta oscillatory power) in the model (see Fig. 5b, lower panel). Nevertheless, together, HDP-related activation and beta oscillatory synchrony in SM1 accounted for 93.3% of the variance in finger tapping performance in a subset PwP completing behavioral testing with triaxial accelerometers (i.e., *N* = 11).

## Discussion

Herein, we used non-invasive, whole-brain MEG recordings to comprehensively quantify proposed neural markers of clinical outcomes relating to STN-DBS parameter efficacy (i.e., directionality and magnitude of currently administered) in a cohort of PwP. Specifically, we observed significant modulations of DBS-induced beta oscillations (i.e., 14–30 Hz), as well as STN-DBS-evoked responses by varying DBS parameters. Together, evoked and induced responses impacted quantitative motor outcomes in a setting-dependent manner, as evidenced by significant neural response x DBS setting interaction in a subset of patients. Below, we discuss the implications of these findings for understanding the comprehensive role of cortical evoked and induced brain-behavior interactions for indexing effective DBS parameters and subsequent clinical outcomes in PwP.

Our findings suggesting that DBS-induced and evoked cortical responses were sensitive to the directionality and magnitude of current applied in the STN were not surprising, as prior work by our lab and others have established this link, albeit for each neurophysiological marker in isolation, rather than in concert within the same experimental cohort. For example, DBS-evoked cortical responses ipsilateral to the site of stimulation tend to be largest when undergoing more clinically effective DBS settings such as optimal contact orientations and larger stimulation amplitudes, albeit the latter may also be concomitant with adverse side effects elicited by the device, including speech disturbances, dizziness, and muscle contractions to name a few^2,3,8,23–25^. In fact, we observed that medium-latency evoked responses tended to scale linearly with increasing stimulation amplitude and more clinically effective contact settings in all relevant sensorimotor areas probed in the current study (i.e., M1, S1, MFG, and SMA).

Similarly, changes in induced cortical activity (i.e., beta synchronization) have been shown to decrease with the application of clinically effective, high-frequency STN-DBS, although these studies have been limited to quantifying ongoing, long-term fluctuations in oscillatory synchrony (e.g., on the order of multiple seconds to minutes)^19,20,26–28^. In contrast, the current study employed a low-frequency monopolar stimulation paradigm (i.e., 2 Hz) during MEG in order to precisely quantify disparate oscillatory profiles immediately following DBS pulse onset. While the application of low-frequency STN-DBS is not effective for improving motor symptoms in PwP, this is a common approach for electrophysiological studies of DBS, with prior studies suggesting a lack of difference in cortical response profiles (e.g., DBS-evoked cortical responses) based on the frequency administered^8^. Thus, we presume the magnitude of DBS parameter-related effects on neural outcomes measured in the current study would follow suit for our cohort and may still result in reliable markers for DBS parameter identification in the future. Importantly, we propose that the aforementioned results reflect neurophysiological correlates of clinical outcomes induced by varying DBS parameters rather than representing the precise mechanism of action of high-frequency stimulation alone, and thus, our results should be interpreted carefully. Nevertheless, we observed significant modulations of induced lower and higher frequency beta oscillatory amplitude (i.e., 14–22 Hz and 24–30 Hz, respectively), but not frequency, in the ipsilateral M1, S1, and SMA, with more effective settings such as best clinical contacts and lower stimulation amplitudes eliciting the greatest reductions in sensorimotor beta power (i.e., weaker beta responses following DBS pulse onset). Of note, our interpretation of lower stimulation amplitudes reflecting better (i.e., more effective) clinical settings is based on the notion that higher stimulation amplitudes (e.g., >4–5 mA) are often coincident with DBS-induced side effects, which limits the clinical efficacy of the setting, even if improvements in motor outcomes are also observed at these higher amplitudes. Interestingly, the magnitude of induced beta power also tended to be weaker for clinically effective contacts at higher stimulation amplitudes compared to higher amplitudes applied at suboptimal contact settings, suggesting a general lack of beta suppression (i.e., reduction in elevated beta synchrony) solely based on the magnitude of current delivered to the STN.

Interestingly, we observed no impact of DBS settings on lower frequency theta-alpha synchronizations (i.e., 4–12 Hz). This effect may implicate divergent disease- and/or symptom-specific modulations of DBS-induced oscillations by varying parameter spaces, as prior electrophysiological studies of DBS suggest that effective DBS protocols for other movement disorder patients (e.g., dystonia) may be conveyed by altered lower frequency activity (i.e., reductions in aberrant theta synchronization), while spectrally-specific modulations in the beta frequency range may be ubiquitous across discrete symptom subtypes regardless of diagnosis (i.e., bradykinesia)^29^. Future studies interrogating such comprehensive DBS-induced oscillatory profiles in other movement disorders or those exhibiting alternative symptoms (e.g., tremors) would help clarify this distinction.

Finally, a central goal of the current study was to probe a well-theorized mechanism of action of STN-DBS, which includes a HDP-related improvement in motor function that may be influenced by the level of beta power also elicited by STN-DBS protocols in animals and humans alike^7,10,12,30^. Indeed, our results demonstrated a full mediation of HDP-related improvements in quantitative motor outcomes through the mediator (i.e., induced sensorimotor beta power) in a subset of PwP who completed standardized movement paradigms with a triaxial accelerometer affixed to their right index finger. Essentially, larger medium-latency evoked responses (i.e., likely reflective of HDP-related activation) in M1 and S1 were significantly predictive of lower, data-driven finger-tapping movement profiles (i.e., better behavioral performance^2^). This relationship also depended on the clinical efficacy of DBS parameters tested, with larger evoked responses predicting better behavior during best contact/clinical amplitude settings, while the opposite trajectory was observed for worst contacts/clinical amplitudes. Importantly, the relationship between evoked response amplitude and behavioral performance was significantly reduced upon the addition of M1 and S1 beta power in the model. Thus, we can conclude that the degree of induced beta synchrony in the ipsilateral primary sensorimotor cortex is an influential and perhaps even causal factor in the DBS-evoked brain–behavior interactions observed in the current cohort. Of note, the quantitative characterization of finger-tapping performance as addressed herein is reflective of data-driven, sample-specific estimations of finger-tapping performance (i.e., single-trial fluctuations in amplitude, speed, movement smoothness, and response variability), which has demonstrated good correspondence with traditional clinical outcomes of general and task-specific motor function (e.g., total MDS-UPDRS-III and Item 3.4 scores^2^, therapeutic windows, see Supplementary Fig. 2) in PwP.

In conclusion, to our knowledge, this is the first study to *comprehensively* quantify the precise evoked *and* induced neurophysiological features pertaining to optimal and suboptimal STN-DBS parameters, including the directionality and magnitude of current administered. Specifically, our results demonstrated temporally- (for evoked analyses) and spectrally-specific (for oscillatory analyses) modulations based on the clinical efficacy of DBS programming strategies, with more effective settings such as clinically useful contacts and stimulation amplitudes eliciting increases in sensorimotor evoked potentials, reductions in elevated sensorimotor beta power, and better behavioral performance on standardized clinical assessments (i.e., accelerometer-based analysis of MDS-UPDRS Item 3.4).

While our results are promising, there are several limitations that should be considered. For example, traditionally, electrophysiological studies of DBS effects employ bipolar stimulation settings instead of clinically useful monopolar ones, in hopes of reducing stimulation-related artifacts (e.g., due to the generator or cable)^31^. However, as the goal of the current study was to interrogate proposed neurophysiological correlates of clinical outcomes, we opted to retain monopolar stimulation settings during MEG to adhere more closely to parameters commonly used in the clinic. To address this, we applied the recommended artifact rejection techniques for simultaneous DBS-MEG recordings for MEGIN systems (i.e., tSSS^31^), and have also avoided potential remnants of the artifact, which may persist less than 2 ms from DBS pulse onset and may cause slight signal leakage in the frequency spectrum. Additionally, while our mediation analysis of brain-behavior interactions was probed in a subset of PwP (*N* = 11) who completed monopolar reviews of finger tapping using wearable sensors, power analyses of observed effects suggest we had moderate to large power to detect each predictive path (i.e., effect size Cohen’s *d* > 0.49–0.81), although future studies interrogating this pathway in larger cohorts will undoubtedly clarify the extent of this pathway for predicting quantitative motor outcomes in PwP. Finally, with the advent of directional leads now allowing for more focal, directed steering of current to the STN and other subcortical structures commonly targeted in DBS therapies (e.g., globus pallidus internus), our primary goal of the current study was to evaluate the impact of best and worst *directional* contact settings on well-theorized neurophysiological and behavioral correlates of clinical outcomes in PwP. However, some individuals are stimulated using traditional, ring-shaped omnidirectional current administration approaches. In a prior study by our laboratory, we systematically tested all directional contacts (i.e., anterior, medial, and laterally-oriented directional contacts), as well as omnidirectional current administration to determine whether DBS-evoked cortical responses and subsequent movement outcomes were modulated based on the orientation of current applied^2^. Indeed, we observed that clinically effective contact settings (regardless of directional or omnidirectional orientation) yielded the largest DBS-evoked cortical responses in the ipsilateral sensorimotor cortex compared to suboptimal contact settings. Moreover, sensorimotor cortical responses evoked by clinically effective contact settings predicted improvements in motor performance on the MDS-UPDRS finger-tapping paradigm. Interestingly, the majority of our prior study’s cohort had omnidirectional or laterally-oriented contacts as their clinically effective contact setting at the time of study enrollment, which was concomitant with higher therapeutic windows and/or reduced side effects than other suboptimal contact settings tested (e.g., anteriorly-oriented directional contacts). Taken together with the results from the current study, we hypothesize that more effective clinical contacts would yield similar trajectories of DBS-evoked and induced cortical responses, as well as quantitative motor outcomes as measured herein, regardless of the nature of the lead itself (i.e., directional vs. omnidirectional current administration). However, future studies will be necessary to clarify this point. Nevertheless, our data suggest that the brain-behavior dynamics (i.e., DBS-evoked and induced cortical responses, accelerometer-based movement outcomes) measured herein may serve as effective targets for guiding optimal DBS parameter selection in PwP, which may, importantly, improve the reliability and efficiency of clinical programming appointments for patients and clinicians alike in the future.

## Methods

### Participant demographics

Twenty PwP (M_age_ = 63.2 years old, 45–76 years old, 4 females) implanted with STN-DBS (Abbott Infinity DBS System, lead model: 6170, Abbott, Plano, Texas, USA) were recruited for this study from the Center for Movement Disorders and Neuromodulation at the University Hospital Düsseldorf. Exclusionary criteria included any medical illness affecting CNS function, any neurological or psychiatric disorder (except PD), severe depression (Beck Depression Inventory > 30), or cognitive impairment (mini-mental state examination < 26). Patients were recorded in their clinically-defined medication state (i.e., ON or OFF dopaminergic medication, depending on their clinical regimen at the time of study enrollment). Of note, only 1 subject was not prescribed dopaminergic medication at the time of study enrollment and was to be recorded in their clinically effective medication OFF state. However, as this subject did not complete all MEG/behavioral aspects of the study, they were excluded from further analyses (see Results below). For a comprehensive description of PD-relevant clinical information, see Table 1. The local ethics committee at Heinrich-Heine University Düsseldorf approved the study (No. 2019–626_2) and all patients provided written informed consent in accordance with the Declaration of Helsinki.

### Monopolar review of best and worst DBS parameter settings

Participants were instructed to complete a finger-tapping paradigm (Item 3.4 of MDS-UPDRS Part-III Examination, respectively) of 10 consecutive movement sequences as largely, quickly, and precisely as possible with their right hand in the air. Simultaneously, we applied monopolar DBS of the left STN at each directional contact (i.e., A, B, and C) at therapeutically beneficial settings (i.e., clinically effective frequency and pulse width, current therapeutic contact height, ≥clinically effective stimulation amplitude). During testing, clinical improvement of bradykinesia symptoms and/or tremor alleviation and immediate, sustained side effects (e.g., dizziness, muscle contractions, speech disturbances) were assessed to determine the best and worst directional contacts based on the size of the therapeutic window (i.e., minimum stimulation amplitude required to elicit clinically relevant benefits vs. side effects, with higher and lower values indicating better or worse contact settings, respectively). For example, contacts eliciting observable alleviations in bradykinesia symptoms (e.g., greater movement amplitudes across the entire task, more regular movement pacing, faster-tapping frequencies) and/or tremor reduction, as well as those with the largest therapeutic window and larger side effect thresholds (i.e., stimulation amplitude in mA eliciting side effects) were chosen as the “best” clinical contact. This also coincided with the directional contact used for their current therapeutic regimen in patients with clinically employed directional leads. In contrast, contacts demonstrating either (1) no observable alleviation in motor symptoms during finger tapping paradigms and/or resting tremor, (2) demonstrating the shortest therapeutic windows, and/or (3) eliciting side effects at lower stimulation amplitudes were determined to be the “worst” directional contact used for subsequent MEG-DBS recordings. Of note, monopolar review testing of best and worst directional contacts and therapeutic effect thresholds was conducted immediately prior to MEG recordings.

### Accelerometry-based quantification of finger-tapping performance

In order to quantify finger-tapping metrics, we developed a novel event detection algorithm using custom-written scripts in MATLAB (Version 2021a)^2^. Finger-tapping blocks were epoched (i.e., ~10 s of consecutive movements) and pre-processed. The acceleration signal was visually inspected for artifacts and filtered using a third-order high-pass Butterworth filter (1 Hz cut-off frequency). Next, probable movement events (i.e., taps) were detected at the single-trial level using a two-stage approach. First, probable movement events were identified using a fixed-threshold algorithm based on the magnitude and jerk (i.e., rate of change of acceleration) percentile thresholds of the accelerometer vector (i.e., 90 and 95th percentiles, respectively). The resulting time windows of probable finger taps (i.e., time of movement onset to offset) were further confirmed using the *findpeaks* function in MATLAB (i.e., minimum peak prominence ≥ 2.5 SD above the accelerometer vector magnitude; minimum peak distance ≥ 100 ms) and supplemented with a visual inspection. The resulting confirmed finger tapping movement events (i.e., single-trial movement onset to offset time windows) were then used to quantify single-trial and grand-averaged behavioral metrics pertinent to MDS-UPDRS rating recommendations including normalized general acceleration magnitude in m/s^2^ (normed to movement duration; i.e., movement onset to offset in ms), inter-tap interval or tap frequency (i.e., peak to peak distance in ms), movement execution smoothness (i.e., acceleration jerk in m/s^3^), and the coefficient of variation of each variable, reflecting the consistency in each metric across the finger tapping block.

### MEG data acquisition and coregistration with structural MRI

All recordings were performed in a three-layer magnetically-shielded room. With an acquisition bandwidth of 0.1–1660 Hz, neuromagnetic responses were sampled continuously at 5 kHz using a MEGIN/Elekta MEG system (MEGIN, Helsinki, Finland) with 306 magnetic sensors, including 204 planar gradiometers and 102 magnetometers. During MEG recordings, patients were instructed to rest with their eyes open and fixated on a crosshair while 2 Hz monopolar stimulation of the left STN was administered so that the inter-pulse interval could be analyzed. Clinically-effective stimulation settings were applied (same as monopolar review above with exception of stimulation frequency) while ~240 stimulation pulses were collected for each contact and stimulation amplitude tested (i.e., a total of six experimental runs lasting approximately 2 min each) in a pseudorandomized order (i.e., A, B, and C directional contacts at clinically-effective stimulation amplitude ± 50%). Prior to MEG acquisition, four coils were attached to the subject’s head and localized, together with fiducial and ~150 scalp surface points, using a three‐dimensional (3D) digitizer (FASTRAK 3SF0002, Polhemus Navigator Sciences, Colchester, Vermont). Throughout data acquisition, participants were monitored using a real-time audio-video feed from inside the magnetically shielded room. MEG data from each patient were subjected to noise reduction using the signal space separation method with a temporal extension^32^. Only data from the gradiometers were used for further analysis. Each participant’s MEG data were coregistered with their pre-surgical structural T1-weighted MRI data prior to imaging analyses using an iterative closest-point rigid-body registration in *Brainstorm*^33^. These fits were manually corrected following visual inspection when appropriate. Structural MRI data were segmented, and cortical surfaces were computed using the CAT12 toolbox in SPM^34^ using the default setting and imported into *Brainstorm*. Individual cortical surfaces were down-sampled to 15,000 vertices for MEG source imaging analyses.

### MEG pre-processing and sensor-level analyses

Cardiac and ocular artifacts were removed from the data using signal-space projection (SSP) and the projection operator was accounted for during source reconstruction^35^. Epochs were of 450 ms duration (i.e., −100 to 350 ms), with 0 ms defined as the onset of the DBS pulse and the baseline being the −100 to −5 ms window. Epochs containing artifacts were rejected based on a fixed threshold method of trial-wise neural amplitude (fT) and gradient (fT/cm/s) values exceeding 3 median absolute deviations, supplemented with visual inspection. On average, 213 ± 14 trials per patient and experimental run (i.e., six experimental conditions testing best and worst directional contacts and clinically effective stimulation amplitudes ± 50%) were used for further analysis. Importantly, the number of trials did not significantly differ as a function of the experimental run (LME: *F*(90) = 1.31, *p* = 0.269).

Artifact-free epochs per patient and experimental run were further processed following two parallel pipelines. For the time-domain (i.e., evoked) analyses, all epochs were averaged with respect to DBS pulse onset for each sensor in the array and normalized to the baseline period. For the oscillatory analyses, epochs were transformed into the time-frequency domain using Morlet wavelets (frequency range: 1–50 Hz; central frequency: 3 Hz; time resolution: 1 s), and the resulting spectral power estimations per sensor were averaged over trials to generate time-frequency plots of mean spectral density. The sensor-level data per time-frequency bin were normalized using the mean power per frequency during the −100 to −5 ms baseline period. The specific time- and time-frequency windows used for evoked and induced source reconstruction pipelines were determined using paired-sample *t*-tests against baseline across subjects, followed up with non-parametric permutation testing to control for multiple comparisons (initial threshold: *p* < 0.05, permutations: 10,000)^36,37^. The permutation procedure used Monte Carlo random sampling to estimate the empirical distribution of the t-statistic at each sensor and time- or time-frequency window in the experimental epoch^36,37^. The resulting evoked and oscillatory activity that significantly differed from baseline (FDR-corrected at *p* < 0.005 and minimum duration of 5 ms for time, frequency, and sensors) was used to guide subsequent evoked and time-frequency domain source-level analyses to select the time- and time-frequency windows of interest. Of note, oscillatory response time windows used for subsequent source analyses were shifted by at least 10 ms surrounding response maxima (i.e., greatest amplitude change from baseline) following DBS pulse onset to avoid remnants of the DBS artifact (i.e., up to 2 ms surrounding DBS pulse onset) and to optimize the signal to noise ratio.

### MEG source imaging

Source images were computed with overlapping spheres head models (15,002 cortical vertices and current flows of constrained orientations) using weighted minimum norm estimation (MNE) using the default parameters in Brainstorm^33^. The noise covariance matrix was obtained from the pre-stimulus period of the experimental epoch. The resulting whole-brain maps were 4-dimensional estimates of current density per vertex, per time sample from −100 to 350 ms locked to DBS pulse onset averaged across all trials. For the oscillatory analysis, these data were transformed into the time-frequency domain per trial using Morlet wavelets and normalized to the baseline period (−100 to −5 ms). The resulting baseline-corrected maps were then projected onto default anatomy for subsequent averaging and statistical modeling. Using the time–frequency clusters identified in the sensor-level analysis, these maps were grand-averaged across all patients, experimental runs and trials to determine the peak vertex per oscillatory response (i.e., theta/alpha: 4–12 Hz from 50–300 ms; low beta: 14–22 Hz from 10–100 ms; high beta: 24–30 Hz from 10–100 ms). From this peak, we computed the relative (i.e., baseline-corrected) response time series of each participant per experimental session across the pre-defined time–frequency window of interest to derive estimates of the induced neural responses (i.e., peak amplitude and frequency) for each participant.

In regard to the time-domain (i.e., evoked) analysis, source images were computed using constrained MNE to produce 4D estimates of current density per vertex and time sample in our experimental epoch. These data were then grand-averaged across patients and experimental conditions to determine the peak cortical responses evoked by STN-DBS. From this peak, baseline-normalized source current density estimates were extracted per experimental condition to derive estimates of the time-domain response for each participant.

### Statistical analyses of neural effects

To examine the influence of DBS parameter settings (i.e., directionality and magnitude of DBS current) on DBS-evoked and induced responses, linear mixed-effects models (LMEs) of experimental session (fixed effect factor with 6 levels—see below) and subject (random effect) on neural response outcomes were conducted separately per evoked and oscillatory response using the *lme4* package in R (Version 4.0.3). Of note, categorical definitions of experimental session (i.e., best and worst directional contacts tested at clinically effective stimulation amplitudes ±50%, see monopolar review above) were factorized with 6 levels (i.e., best contact, low amplitude; best contact, clinical amplitude; best contact, high amplitude; worst contact, low amplitude; worst contact, clinical amplitude; worst contact, high amplitude). Importantly, all LME post-hoc analyses were corrected for multiple comparisons using Tukey’s multiple comparison test.

### Quantitative analysis of brain–behavior interactions

To date, few studies have investigated the comprehensive impact of proposed neurophysiological features of STN-DBS (e.g., stimulation-evoked cortical responses, beta suppression) on clinical outcomes in PwP. Given the recent literature proposing a link between STN-related hyperdirect pathway activation (i.e., HDP: DBS-evoked cortical responses ~3–10 ms following DBS pulse onset), cortical beta oscillations, and motor function in model systems, we aimed to evaluate the relationship between cortical beta power and HDP-related improvements in motor function using mediation analyses^38^. Specifically, we hypothesized a full mediation of quantitative motor outcomes (see below) by stimulation-evoked cortical activations in sensorimotor cortices (i.e., primary motor and somatosensory cortices: SM1) through the mediator (i.e., SM1 beta power).

To quantitatively characterize motor function, a subset of PwP (*N* = 11) who completed MDS-UPDRS Item 3.4 finger tapping protocols with a triaxial accelerometer (ADXL335 iMEMs Accelerometer, Analog Devices, Inc., Norwood, MA, USA) attached to the right index finger were assessed. Importantly, all participants were tested in the clinically defined medication ON state, as well as clinically-effective stimulation ON state (i.e., 130 Hz stimulation frequency, clinically effective contact height, best and worst directional contacts (see monopolar review above), clinically effective stimulation amplitude and pulse width). These recordings were completed outside of the MEG scanner. Specifically, to index relevant metrics of finger tapping movement profiles, we conducted an exploratory factor analysis (EFA) to define a single component of movement using a compilation of accelerometer metrics known to be reflective of DBS therapy-related fluctuations in motor outcomes (i.e., acceleration magnitude and variability, movement execution smoothness and variability, inter-tap interval or movement pacing and variability). Further details on our accelerometer processing pipeline can be found in recent papers^2,3^. We began with a set list of six measures and progressively removed individual variables based on poor loadings (*λ* < 0.70) and overall model fit^2,39^. The best-fitting model was used to define a latent variable for which a movement profile score was extracted per participant. Modeling and component extraction were completed using *lavaan* and *principal* functions in R, respectively. Of note, lower finger-tapping movement profile scores are reflective of smoother finger-tapping movements, and each score was subsequently extracted per participant and experimental session and entered as dependent variables in our regression-based mediation analyses using LMEs. Specifically, we probed the differential impact on finger tapping movement profiles as a function of HDP-related activation (i.e., DBS-evoked cortical responses ~3–7 ms; continuous variable), experimental session (factor with 2 levels: best and worst contact at clinical amplitudes) and their interaction with and without the mediator (i.e., DBS-induced beta power 14–30 Hz) controlling for subject (factor with 11 levels) and ROI/frequency (factor with 3 levels: M1 low beta, S1 low and high beta power) as a nested random effect.

Finally, therapeutic windows for best and worst contact settings are reported for the current sample and were related to experimental session and quantitative movement profile scores using LMEs, separately as a descriptive comparison to the neurophysiological and behavioral data reported herein (see Supplementary Materials).

### Reporting summary

Further information on research design is available in the Nature Research Reporting Summary linked to this article.

### Supplementary information

Supplementary Material

Reporting Summary

## Data Availability

The anonymized data from this study will be made available to investigators upon request from the corresponding authors.

## Code availability

The code used in this study will be made available to investigators upon request from the corresponding authors.
