# Supplementary material for: Modulation of DBS-induced cortical responses and movement by the directionality and magnitude of current administered
Source: NPJ Parkinsons Dis. 2024 Mar 8;10:53. doi: 10.1038/s41531-024-00663-9 (PMC10923868; doi:10.1038/s41531-024-00663-9)
Supplement: Supplementary file 1 — Supplementary Material [file 41531_2024_663_MOESM1_ESM.docx]

**Supplementary Materials**

**Supplementary Table 1. Model results of experimental session on M1 theta-alpha induced response amplitude during STN-DBS.**

| ***M1 Theta-Alpha Induced Response Amplitude*** | | | | | |
| --- | --- | --- | --- | --- | --- |
| **Effect** | **F** | **p** | **Effect Size (d)** | **Lower CI** | **Upper CI** |
| Experimental Session | 0.80 | 0.555 | 0.19 | -0.23 | 0.61 |

*Note.* Cohen’s d effect sizes and associated 95% confidence intervals (CI) were calculated based on appropriate test statistics using the *effectsize* package in R. Bolded values indicate statistical significance and trending effects based on *p_corrected_* < .05 and .10, respectively following Tukey’s correction for multiple comparisons and non-overlapping confidence intervals of effect sizes.

**Supplementary Table 2. Model results of experimental session on S1 theta-alpha induced response amplitude during STN-DBS.**

| ***S1 Theta-Alpha Induced Response Amplitude*** | | | | | |
| --- | --- | --- | --- | --- | --- |
| **Effect** | **F** | **p** | **Effect Size (d)** | **Lower CI** | **Upper CI** |
| Experimental Session | 1.53 | 0.190 | 0.28 | -0.17 | 0.73 |

*Note.* Cohen’s d effect sizes and associated 95% confidence intervals (CI) were calculated based on appropriate test statistics using the *effectsize* package in R. Bolded values indicate statistical significance and trending effects based on *p_corrected_* < .05 and .10, respectively following Tukey’s correction for multiple comparisons and non-overlapping confidence intervals of effect sizes.

**Supplementary Table 3. Model results of experimental session on MFG theta-alpha induced response amplitude during STN-DBS.**

| ***MFG Theta-Alpha Induced Response Amplitude*** | | | | | |
| --- | --- | --- | --- | --- | --- |
| **Effect** | **F** | **p** | **Effect Size (d)** | **Lower CI** | **Upper CI** |
| Experimental Session | 1.17 | 0.328 | 0.23 | -0.19 | 0.65 |

*Note.* Cohen’s d effect sizes and associated 95% confidence intervals (CI) were calculated based on appropriate test statistics using the *effectsize* package in R. Bolded values indicate statistical significance and trending effects based on *p_corrected_* < .05 and .10, respectively following Tukey’s correction for multiple comparisons and non-overlapping confidence intervals of effect sizes.

**Supplementary Table 4. Model results of experimental session on SMA theta-alpha induced response amplitude during STN-DBS.**

| ***SMA Theta-Alpha Induced Response Amplitude*** | | | | | |
| --- | --- | --- | --- | --- | --- |
| **Effect** | **F** | **p** | **Effect Size (d)** | **Lower CI** | **Upper CI** |
| Experimental Session | 1.63 | 0.160 | 0.27 | -0.15 | 0.70 |

*Note.* Cohen’s d effect sizes and associated 95% confidence intervals (CI) were calculated based on appropriate test statistics using the *effectsize* package in R. Bolded values indicate statistical significance and trending effects based on *p_corrected_* < .05 and .10, respectively following Tukey’s correction for multiple comparisons and non-overlapping confidence intervals of effect sizes.

**Supplementary Table 5. Model results of experimental session on M1 low beta induced response amplitude during STN-DBS.**

| ***M1 Low Beta Induced Response Amplitude*** | | | | | |
| --- | --- | --- | --- | --- | --- |
| **Effect** | **F** | **p** | **Effect Size (d)** | **Lower CI** | **Upper CI** |
| Experimental Session | 3.41 | **0.007** | 0.39 | -0.03 | 0.81 |
| ***Post Hoc Testing*** | | | | | |
| **Main Effect of Experimental Session** | **t** | **p** | **Effect Size (d)** | **Lower CI** | **Upper CI** |
| 1 vs 2 | -0.71 | 0.479 | -0.08 | -0.28 | 0.13 |
| 1 vs 3 | -2.25 | **0.027** | -0.24 | -0.45 | -0.03 |
| 1 vs 4 | -0.34 | 0.734 | -0.04 | -0.25 | 0.17 |
| 1 vs 5 | -0.73 | 0.466 | -0.08 | -0.29 | 0.13 |
| 1 vs 6 | -3.40 | **0.001** | -0.36 | -0.58 | -0.15 |
| 2 vs 3 | -1.54 | 0.127 | -0.16 | -0.37 | 0.05 |
| 2 vs 4 | 0.37 | 0.712 | 0.04 | -0.17 | 0.25 |
| 2 vs 5 | -0.02 | 0.982 | 0.00 | -0.21 | 0.21 |
| 2 vs 6 | -2.70 | **0.008** | -0.29 | -0.50 | -0.07 |
| 3 vs 4 | 1.91 | 0.059 | 0.20 | -0.01 | 0.41 |
| 3 vs 5 | 1.52 | 0.132 | 0.16 | -0.05 | 0.37 |
| 3 vs 6 | -1.19 | 0.239 | -0.13 | -0.34 | 0.08 |
| 4 vs 5 | -0.39 | 0.696 | -0.04 | -0.25 | 0.17 |
| 4 vs 6 | -3.07 | **0.003** | -0.33 | -0.54 | -0.11 |
| 5 vs 6 | -2.68 | **0.009** | -0.29 | -0.50 | -0.07 |
| ***Estimated Marginal Means (EMM)*** | | | | | |
| **Experimental Session** | | **EMM (%)** | **SE** | **Lower CI** | **Upper CI** |
| Best Contact, Low Amplitude | | 71.60 | 15.90 | 39.90 | 103.00 |
| Best Contact, Clinical Amplitude | | 84.00 | 15.90 | 52.30 | 116.00 |
| Best Contact, High Amplitude | | 111.00 | 15.90 | 79.30 | 143.00 |
| Worst Contact, Low Amplitude | | 77.50 | 15.90 | 45.80 | 109.00 |
| Worst Contact, Clinical Amplitude | | 84.40 | 15.90 | 52.70 | 116.00 |
| Worst Contact, High Amplitude | | 132.00 | 16.20 | 99.80 | 164.00 |

*Note.* Cohen’s d effect sizes and associated 95% confidence intervals (CI) were calculated based on appropriate test statistics using the *effectsize* package in R. Bolded values indicate statistical significance and trending effects based on *p_corrected_* < .05 and .10, respectively following Tukey’s correction for multiple comparisons and non-overlapping confidence intervals of effect sizes. **p_corrected_* < .001. 1 = best contact, low amplitude; 2 = best contact, clinical amplitude; 3 = best contact, high amplitude; 4 = worst contact, low amplitude; 5 = worst contact, clinical amplitude; 6 = worst contact, high amplitude.

**Supplementary Table 6. Model results of experimental session on S1 low beta induced response amplitude during STN-DBS.**

| ***S1 Low Beta Induced Response Amplitude*** | | | | | |
| --- | --- | --- | --- | --- | --- |
| **Effect** | **F** | **p** | **Effect Size (d)** | **Lower CI** | **Upper CI** |
| Experimental Session | 2.68 | **0.026** | 0.35 | 0.07 | 0.77 |
| ***Post Hoc Testing*** | | | | | |
| **Main Effect of Experimental Session** | **t** | **p** | **Effect Size (d)** | **Lower CI** | **Upper CI** |
| 1 vs 2 | -0.14 | 0.889 | -0.02 | -0.24 | 0.21 |
| 1 vs 3 | -1.31 | 0.193 | -0.15 | -0.37 | 0.08 |
| 1 vs 4 | -0.29 | 0.774 | -0.03 | -0.25 | 0.19 |
| 1 vs 5 | -0.19 | 0.849 | -0.02 | -0.24 | 0.20 |
| 1 vs 6 | -3.00 | **0.003** | -0.34 | -0.57 | -0.11 |
| 2 vs 3 | -1.17 | 0.243 | -0.13 | -0.35 | 0.09 |
| 2 vs 4 | -0.15 | 0.883 | -0.02 | -0.24 | 0.21 |
| 2 vs 5 | -0.05 | 0.960 | -0.01 | -0.23 | 0.22 |
| 2 vs 6 | -2.86 | **0.005** | -0.32 | -0.55 | -0.10 |
| 3 vs 4 | 1.03 | 0.306 | 0.12 | -0.11 | 0.34 |
| 3 vs 5 | 1.13 | 0.263 | 0.13 | -0.10 | 0.35 |
| 3 vs 6 | -1.67 | 0.264 | -0.19 | -0.41 | 0.04 |
| 4 vs 5 | 0.10 | 0.099 | 0.01 | -0.21 | 0.23 |
| 4 vs 6 | -2.72 | **0.008** | -0.31 | -0.53 | -0.08 |
| 5 vs 6 | -2.81 | **0.006** | -0.32 | -0.54 | -0.09 |
| ***Estimated Marginal Means (EMM)*** | | | | | |
| **Experimental Session** | | **EMM (%)** | **SE** | **Lower CI** | **Upper CI** |
| Best Contact, Low Amplitude | | 72.00 | 12.90 | 46.30 | 97.70 |
| Best Contact, Clinical Amplitude | | 74.20 | 12.90 | 48.50 | 99.90 |
| Best Contact, High Amplitude | | 92.60 | 13.30 | 66.30 | 119.00 |
| Worst Contact, Low Amplitude | | 76.50 | 12.90 | 50.80 | 102.20 |
| Worst Contact, Clinical Amplitude | | 75.00 | 2.90 | 49.30 | 100.70 |
| Worst Contact, High Amplitude | | 119.10 | 13.30 | 92.70 | 145.40 |

*Note.* Cohen’s d effect sizes and associated 95% confidence intervals (CI) were calculated based on appropriate test statistics using the *effectsize* package in R. Bolded values indicate statistical significance and trending effects based on *p_corrected_* < .05 and .10, respectively following Tukey’s correction for multiple comparisons and non-overlapping confidence intervals of effect sizes. **p_corrected_* < .001. 1 = best contact, low amplitude; 2 = best contact, clinical amplitude; 3 = best contact, high amplitude; 4 = worst contact, low amplitude; 5 = worst contact, clinical amplitude; 6 = worst contact, high amplitude.

**Supplementary Table 7. Model results of experimental session on MFG low beta induced response amplitude during STN-DBS.**

| ***MFG Low Beta Induced Response Amplitude*** | | | | | |
| --- | --- | --- | --- | --- | --- |
| **Effect** | **F** | **p** | **Effect Size (d)** | **Lower CI** | **Upper CI** |
| Experimental Session | 1.83 | 0.116 | 0.29 | -0.13 | 0.71 |

*Note.* Cohen’s d effect sizes and associated 95% confidence intervals (CI) were calculated based on appropriate test statistics using the *effectsize* package in R. Bolded values indicate statistical significance and trending effects based on *p_corrected_* < .05 and .10, respectively following Tukey’s correction for multiple comparisons and non-overlapping confidence intervals of effect sizes.

**Supplementary Table 8. Model results of experimental session on SMA low beta induced response amplitude during STN-DBS.**

| ***SMA Low Beta Induced Response Amplitude*** | | | | | |
| --- | --- | --- | --- | --- | --- |
| **Effect** | **F** | **p** | **Effect Size (d)** | **Lower CI** | **Upper CI** |
| Experimental Session | 0.84 | 0.526 | 0.23 | -0.22 | 0.67 |

*Note.* Cohen’s d effect sizes and associated 95% confidence intervals (CI) were calculated based on appropriate test statistics using the *effectsize* package in R. Bolded values indicate statistical significance and trending effects based on *p_corrected_* < .05 and .10, respectively following Tukey’s correction for multiple comparisons and non-overlapping confidence intervals of effect sizes.

**Supplementary Table 9. Model results of experimental session on M1 high beta induced response amplitude during STN-DBS.**

| ***M1 High Beta Induced Response Amplitude*** | | | | | |
| --- | --- | --- | --- | --- | --- |
| **Effect** | **F** | **p** | **Effect Size (d)** | **Lower CI** | **Upper CI** |
| Experimental Session | 0.89 | 0.491 | 0.20 | -0.22 | 0.62 |

*Note.* Cohen’s d effect sizes and associated 95% confidence intervals (CI) were calculated based on appropriate test statistics using the *effectsize* package in R. Bolded values indicate statistical significance and trending effects based on *p_corrected_* < .05 and .10, respectively following Tukey’s correction for multiple comparisons and non-overlapping confidence intervals of effect sizes.

**Supplementary Table 10. Model results of experimental session on S1 high beta induced response amplitude during STN-DBS.**

| ***S1 High Beta Induced Response Amplitude*** | | | | | |
| --- | --- | --- | --- | --- | --- |
| **Effect** | **F** | **p** | **Effect Size (d)** | **Lower CI** | **Upper CI** |
| Experimental Session | 3.73 | **0.004** | 0.41 | 0.00 | 0.84 |
| ***Post Hoc Testing*** | | | | | |
| **Main Effect of Experimental Session** | **t** | **p** | **Effect Size (d)** | **Lower CI** | **Upper CI** |
| 1 vs 2 | -3.35 | **0.001** | -0.36 | -0.57 | -0.14 |
| 1 vs 3 | -2.87 | **0.005** | -0.31 | -0.52 | -0.09 |
| 1 vs 4 | -2.29 | **0.024** | -0.25 | -0.46 | -0.03 |
| 1 vs 5 | -3.67 | **0.001*** | -0.39 | -0.61 | -0.17 |
| 1 vs 6 | -3.38 | **0.001** | -0.36 | -0.58 | -0.14 |
| 2 vs 3 | 0.47 | 0.636 | 0.05 | -0.16 | 0.26 |
| 2 vs 4 | 1.10 | 0.276 | 0.12 | -0.09 | 0.33 |
| 2 vs 5 | -0.31 | 0.755 | -0.03 | -0.24 | 0.18 |
| 2 vs 6 | -0.03 | 0.975 | 0.00 | -0.21 | 0.21 |
| 3 vs 4 | 0.62 | 0.539 | 0.07 | -0.14 | 0.28 |
| 3 vs 5 | -0.79 | 0.433 | -0.08 | -0.29 | 0.13 |
| 3 vs 6 | -0.51 | 0.614 | -0.05 | -0.26 | 0.16 |
| 4 vs 5 | -1.41 | 0.161 | -0.15 | -0.36 | 0.06 |
| 4 vs 6 | -1.13 | 0.262 | -0.12 | -0.33 | 0.09 |
| 5 vs 6 | 0.28 | 0.779 | 0.03 | -0.18 | 0.24 |
| ***Estimated Marginal Means (EMM)*** | | | | | |
| **Experimental Session** | | **EMM (%)** | **SE** | **Lower CI** | **Upper CI** |
| Best Contact, Low Amplitude | | 114.00 | 15.30 | 83.60 | 114.00 |
| Best Contact, Clinical Amplitude | | 175.00 | 15.70 | 143.40 | 206.00 |
| Best Contact, High Amplitude | | 166.00 | 15.70 | 134.70 | 197.00 |
| Worst Contact, Low Amplitude | | 155.00 | 15.30 | 124.40 | 185.00 |
| Worst Contact, Clinical Amplitude | | 180.00 | 15.70 | 149.10 | 211.00 |
| Worst Contact, High Amplitude | | 175.00 | 15.70 | 144.00 | 206.00 |

*Note.* Cohen’s d effect sizes and associated 95% confidence intervals (CI) were calculated based on appropriate test statistics using the *effectsize* package in R. Bolded values indicate statistical significance and trending effects based on *p_corrected_* < .05 and .10, respectively following Tukey’s correction for multiple comparisons and non-overlapping confidence intervals of effect sizes. **p_corrected_* < .001. 1 = best contact, low amplitude; 2 = best contact, clinical amplitude; 3 = best contact, high amplitude; 4 = worst contact, low amplitude; 5 = worst contact, clinical amplitude; 6 = worst contact, high amplitude.

**Supplementary Table 11. Model results of experimental session on MFG high beta induced response amplitude during STN-DBS.**

| ***MFG High Beta Induced Response Amplitude*** | | | | | |
| --- | --- | --- | --- | --- | --- |
| **Effect** | **F** | **p** | **Effect Size (d)** | **Lower CI** | **Upper CI** |
| Experimental Session | 1.59 | 0.172 | 0.27 | -0.15 | 0.69 |

*Note.* Cohen’s d effect sizes and associated 95% confidence intervals (CI) were calculated based on appropriate test statistics using the *effectsize* package in R. Bolded values indicate statistical significance and trending effects based on *p_corrected_* < .05 and .10, respectively following Tukey’s correction for multiple comparisons and non-overlapping confidence intervals of effect sizes. **p_corrected_* < .001. 1 = best contact, low amplitude; 2 = best contact, clinical amplitude; 3 = best contact, high amplitude; 4 = worst contact, low amplitude; 5 = worst contact, clinical amplitude; 6 = worst contact, high amplitude.

**Supplementary Table 12. Model results of experimental session on SMA high beta induced response amplitude during STN-DBS.**

| ***SMA High Beta Induced Response Amplitude*** | | | | | |
| --- | --- | --- | --- | --- | --- |
| **Effect** | **F** | **p** | **Effect Size (d)** | **Lower CI** | **Upper CI** |
| Experimental Session | 2.63 | **0.029** | 0.35 | 0.08 | 0.77 |
| ***Post Hoc Testing*** | | | | | |
| **Main Effect of Experimental Session** | **t** | **p** | **Effect Size (d)** | **Lower CI** | **Upper CI** |
| 1 vs 2 | -1.25 | 0.214 | -0.13 | -0.35 | 0.08 |
| 1 vs 3 | -1.16 | 0.251 | -0.12 | -0.34 | 0.09 |
| 1 vs 4 | 0.17 | 0.869 | 0.02 | -0.19 | 0.23 |
| 1 vs 5 | -2.17 | **0.033** | -0.23 | -0.45 | -0.02 |
| 1 vs 6 | -2.73 | **0.008** | -0.29 | -0.51 | -0.08 |
| 2 vs 3 | 0.10 | 0.924 | 0.01 | -0.20 | 0.22 |
| 2 vs 4 | 1.42 | 0.160 | 0.15 | -0.06 | 0.36 |
| 2 vs 5 | -0.90 | 0.368 | -0.10 | -0.31 | 0.11 |
| 2 vs 6 | -1.46 | 0.148 | -0.16 | -0.37 | 0.06 |
| 3 vs 4 | 1.32 | 0.190 | 0.14 | -0.07 | 0.35 |
| 3 vs 5 | -1.00 | 0.319 | -0.11 | -0.32 | 0.10 |
| 3 vs 6 | -1.55 | 0.124 | -0.17 | -0.38 | 0.05 |
| 4 vs 5 | -2.33 | **0.022** | -0.25 | -0.46 | -0.04 |
| 4 vs 6 | -2.89 | **0.005** | -0.31 | -0.52 | -0.09 |
| 5 vs 6 | -0.55 | 0.583 | -0.06 | -0.27 | 0.15 |
| ***Estimated Marginal Means (EMM)*** | | | | | |
| **Experimental Session** | | **EMM (%)** | **SE** | **Lower CI** | **Upper CI** |
| Best Contact, Low Amplitude | | 132.00 | 10.10 | 113.00 | 152.00 |
| Best Contact, Clinical Amplitude | | 150.00 | 10.40 | 129.00 | 171.00 |
| Best Contact, High Amplitude | | 149.00 | 10.40 | 128.00 | 169.00 |
| Worst Contact, Low Amplitude | | 130.00 | 10.10 | 110.00 | 150.00 |
| Worst Contact, Clinical Amplitude | | 163.00 | 10.40 | 142.00 | 183.00 |
| Worst Contact, High Amplitude | | 171.00 | 10.40 | 150.00 | 191.00 |

*Note.* Cohen’s d effect sizes and associated 95% confidence intervals (CI) were calculated based on appropriate test statistics using the *effectsize* package in R. Bolded values indicate statistical significance and trending effects based on *p_corrected_* < .05 and .10, respectively following Tukey’s correction for multiple comparisons and non-overlapping confidence intervals of effect sizes. **p_corrected_* < .001. 1 = best contact, low amplitude; 2 = best contact, clinical amplitude; 3 = best contact, high amplitude; 4 = worst contact, low amplitude; 5 = worst contact, clinical amplitude; 6 = worst contact, high amplitude.

**Supplementary Table 13.** **Model results of experimental session on M1 low beta peak frequency during STN-DBS.**

| ***M1 Low Beta Peak Frequency*** | | | | | |
| --- | --- | --- | --- | --- | --- |
| **Effect** | **F** | **p** | **Effect Size (d)** | **Lower CI** | **Upper CI** |
| Experimental Session | 0.17 | 0.972 | 0.09 | -0.33 | 0.51 |

*Note.* Cohen’s d effect sizes and associated 95% confidence intervals (CI) were calculated based on appropriate test statistics using the *effectsize* package in R. Bolded values indicate statistical significance and trending effects based on *p_corrected_* < .05 and .10, respectively following Tukey’s correction for multiple comparisons and non-overlapping confidence intervals of effect sizes. **p_corrected_* < .001. 1 = best contact, low amplitude; 2 = best contact, clinical amplitude; 3 = best contact, high amplitude; 4 = worst contact, low amplitude; 5 = worst contact, clinical amplitude; 6 = worst contact, high amplitude.

**Supplementary Table 14.** **Model results of experimental session on S1 low beta peak frequency during STN-DBS.**

| ***S1 Low Beta Peak Frequency*** | | | | | |
| --- | --- | --- | --- | --- | --- |
| **Effect** | **F** | **p** | **Effect Size (d)** | **Lower CI** | **Upper CI** |
| Experimental Session | 0.45 | 0.815 | 0.14 | -0.28 | 0.56 |

*Note.* Cohen’s d effect sizes and associated 95% confidence intervals (CI) were calculated based on appropriate test statistics using the *effectsize* package in R. Bolded values indicate statistical significance and trending effects based on *p_corrected_* < .05 and .10, respectively following Tukey’s correction for multiple comparisons and non-overlapping confidence intervals of effect sizes. **p_corrected_* < .001. 1 = best contact, low amplitude; 2 = best contact, clinical amplitude; 3 = best contact, high amplitude; 4 = worst contact, low amplitude; 5 = worst contact, clinical amplitude; 6 = worst contact, high amplitude.

**Supplementary Table 15.** **Model results of experimental session on MFG low beta peak frequency during STN-DBS.**

| ***MFG Low Beta Peak Frequency*** | | | | | |
| --- | --- | --- | --- | --- | --- |
| **Effect** | **F** | **p** | **Effect Size (d)** | **Lower CI** | **Upper CI** |
| Experimental Session | 0.96 | 0.447 | 0.19 | -0.19 | 0.57 |

*Note.* Cohen’s d effect sizes and associated 95% confidence intervals (CI) were calculated based on appropriate test statistics using the *effectsize* package in R. Bolded values indicate statistical significance and trending effects based on *p_corrected_* < .05 and .10, respectively following Tukey’s correction for multiple comparisons and non-overlapping confidence intervals of effect sizes. **p_corrected_* < .001. 1 = best contact, low amplitude; 2 = best contact, clinical amplitude; 3 = best contact, high amplitude; 4 = worst contact, low amplitude; 5 = worst contact, clinical amplitude; 6 = worst contact, high amplitude.

**Supplementary Table 16.** **Model results of experimental session on SMA low beta peak frequency during STN-DBS.**

| ***SMA Low Beta Peak Frequency*** | | | | | |
| --- | --- | --- | --- | --- | --- |
| **Effect** | **F** | **p** | **Effect Size (d)** | **Lower CI** | **Upper CI** |
| Experimental Session | 1.13 | 0.351 | 0.20 | -0.17 | 0.58 |

*Note.* Cohen’s d effect sizes and associated 95% confidence intervals (CI) were calculated based on appropriate test statistics using the *effectsize* package in R. Bolded values indicate statistical significance and trending effects based on *p_corrected_* < .05 and .10, respectively following Tukey’s correction for multiple comparisons and non-overlapping confidence intervals of effect sizes. **p_corrected_* < .001. 1 = best contact, low amplitude; 2 = best contact, clinical amplitude; 3 = best contact, high amplitude; 4 = worst contact, low amplitude; 5 = worst contact, clinical amplitude; 6 = worst contact, high amplitude.

**Supplementary Table 17.** **Model results of experimental session on M1 high beta peak frequency during STN-DBS.**

| ***M1 High Beta Peak Frequency*** | | | | | |
| --- | --- | --- | --- | --- | --- |
| **Effect** | **F** | **p** | **Effect Size (d)** | **Lower CI** | **Upper CI** |
| Experimental Session | 0.80 | 0.552 | 0.19 | -0.23 | 0.61 |

*Note.* Cohen’s d effect sizes and associated 95% confidence intervals (CI) were calculated based on appropriate test statistics using the *effectsize* package in R. Bolded values indicate statistical significance and trending effects based on *p_corrected_* < .05 and .10, respectively following Tukey’s correction for multiple comparisons and non-overlapping confidence intervals of effect sizes. **p_corrected_* < .001. 1 = best contact, low amplitude; 2 = best contact, clinical amplitude; 3 = best contact, high amplitude; 4 = worst contact, low amplitude; 5 = worst contact, clinical amplitude; 6 = worst contact, high amplitude.

**Supplementary Table 18.** **Model results of experimental session on S1 high beta peak frequency during STN-DBS.**

| ***S1 High Beta Peak Frequency*** | | | | | |
| --- | --- | --- | --- | --- | --- |
| **Effect** | **F** | **p** | **Effect Size (d)** | **Lower CI** | **Upper CI** |
| Experimental Session | 1.63 | 0.161 | 0.27 | -0.15 | 0.69 |

*Note.* Cohen’s d effect sizes and associated 95% confidence intervals (CI) were calculated based on appropriate test statistics using the *effectsize* package in R. Bolded values indicate statistical significance and trending effects based on *p_corrected_* < .05 and .10, respectively following Tukey’s correction for multiple comparisons and non-overlapping confidence intervals of effect sizes. **p_corrected_* < .001. 1 = best contact, low amplitude; 2 = best contact, clinical amplitude; 3 = best contact, high amplitude; 4 = worst contact, low amplitude; 5 = worst contact, clinical amplitude; 6 = worst contact, high amplitude.

**Supplementary Table 19.** **Model results of experimental session on MFG high beta peak frequency during STN-DBS.**

| ***MFG High Beta Peak Frequency*** | | | | | |
| --- | --- | --- | --- | --- | --- |
| **Effect** | **F** | **p** | **Effect Size (d)** | **Lower CI** | **Upper CI** |
| Experimental Session | 1.59 | 0.172 | 0.16 | -0.26 | 0.58 |

*Note.* Cohen’s d effect sizes and associated 95% confidence intervals (CI) were calculated based on appropriate test statistics using the *effectsize* package in R. Bolded values indicate statistical significance and trending effects based on *p_corrected_* < .05 and .10, respectively following Tukey’s correction for multiple comparisons and non-overlapping confidence intervals of effect sizes. **p_corrected_* < .001. 1 = best contact, low amplitude; 2 = best contact, clinical amplitude; 3 = best contact, high amplitude; 4 = worst contact, low amplitude; 5 = worst contact, clinical amplitude; 6 = worst contact, high amplitude.

**Supplementary Table 20.** **Model results of experimental session on SMA high beta peak frequency during STN-DBS.**

| ***SMA High Beta Peak Frequency*** | | | | | |
| --- | --- | --- | --- | --- | --- |
| **Effect** | **F** | **p** | **Effect Size (d)** | **Lower CI** | **Upper CI** |
| Experimental Session | 2.29 | 0.052 | 0.32 | -0.10 | 0.74 |

*Note.* Cohen’s d effect sizes and associated 95% confidence intervals (CI) were calculated based on appropriate test statistics using the *effectsize* package in R. Bolded values indicate statistical significance and trending effects based on *p_corrected_* < .05 and .10, respectively following Tukey’s correction for multiple comparisons and non-overlapping confidence intervals of effect sizes. **p_corrected_* < .001. 1 = best contact, low amplitude; 2 = best contact, clinical amplitude; 3 = best contact, high amplitude; 4 = worst contact, low amplitude; 5 = worst contact, clinical amplitude; 6 = worst contact, high amplitude.

**Supplementary Table 21.** **Model results of experimental session on M1 medium-latency evoked response amplitude during STN-DBS.**

| ***M1 Medium-Latency Evoked Response Amplitude*** | | | | | |
| --- | --- | --- | --- | --- | --- |
| **Effect** | **F** | **p** | **Effect Size (d)** | **Lower CI** | **Upper CI** |
| Experimental Session | 12.52 | **0.001*** | 0.41 | 0.18 | 0.64 |
| ***Post Hoc Testing*** | | | | | |
| **Main Effect of Experimental Session** | **t** | **p** | **Effect Size (d)** | **Lower CI** | **Upper CI** |
| 1 vs 2 | -2.18 | **0.030** | -0.13 | -0.24 | -0.01 |
| 1 vs 3 | -1.75 | 0.081 | -0.10 | -0.22 | 0.01 |
| 1 vs 4 | 0.03 | 0.973 | 0.00 | -0.11 | 0.12 |
| 1 vs 5 | -2.35 | **0.020** | -0.14 | -0.25 | -0.02 |
| 1 vs 6 | -6.89 | **0.001*** | -0.40 | -0.52 | -0.28 |
| 2 vs 3 | 0.39 | 0.696 | 0.02 | -0.09 | 0.14 |
| 2 vs 4 | 2.18 | **0.030** | 0.13 | 0.01 | 0.24 |
| 2 vs 5 | -0.17 | 0.869 | -0.01 | -0.12 | 0.10 |
| 2 vs 6 | -4.74 | **0.001*** | -0.28 | -0.39 | -0.16 |
| 3 vs 4 | 1.76 | 0.080 | 0.10 | -0.01 | 0.22 |
| 3 vs 5 | -0.55 | 0.580 | -0.03 | -0.15 | 0.08 |
| 3 vs 6 | -5.05 | **0.001*** | -0.29 | -0.41 | -0.18 |
| 4 vs 5 | -2.34 | **0.020** | -0.14 | -0.25 | -0.02 |
| 4 vs 6 | -6.81 | **0.001*** | -0.40 | -0.51 | -0.28 |
| 5 vs 6 | -4.58 | **0.001*** | -0.27 | -0.38 | -0.15 |
| ***Estimated Marginal Means (EMM)*** | | | | | |
| **Experimental Session** | | **EMM (%)** | **SE** | **Lower CI** | **Upper CI** |
| Best Contact, Low Amplitude | | 4.23 | 0.48 | 3.27 | 5.20 |
| Best Contact, Clinical Amplitude | | 5.29 | 0.48 | 4.32 | 6.25 |
| Best Contact, High Amplitude | | 5.09 | 0.49 | 4.11 | 6.08 |
| Worst Contact, Low Amplitude | | 4.22 | 0.49 | 3.23 | 5.20 |
| Worst Contact, Clinical Amplitude | | 5.36 | 0.48 | 4.40 | 6.33 |
| Worst Contact, High Amplitude | | 7.61 | 0.49 | 6.63 | 8.60 |

*Note.* Cohen’s d effect sizes and associated 95% confidence intervals (CI) were calculated based on appropriate test statistics using the *effectsize* package in R. Bolded values indicate statistical significance and trending effects based on *p_corrected_* < .05 and .10, respectively following Tukey’s correction for multiple comparisons and non-overlapping confidence intervals of effect sizes. **p_corrected_* < .001. 1 = best contact, low amplitude; 2 = best contact, clinical amplitude; 3 = best contact, high amplitude; 4 = worst contact, low amplitude; 5 = worst contact, clinical amplitude; 6 = worst contact, high amplitude.

**Supplementary Table 22.** **Model results of experimental session on S1 medium-latency evoked response amplitude during STN-DBS.**

| ***S1 Medium-Latency Evoked Response Amplitude*** | | | | | |
| --- | --- | --- | --- | --- | --- |
| **Effect** | **F** | **p** | **Effect Size (d)** | **Lower CI** | **Upper CI** |
| Experimental Session | 18.85 | **0.001*** | 0.50 | 0.27 | 0.74 |
| ***Post Hoc Testing*** | | | | | |
| **Main Effect of Experimental Session** | **t** | **p** | **Effect Size (d)** | **Lower CI** | **Upper CI** |
| 1 vs 2 | -0.32 | 0.747 | -0.02 | -0.13 | 0.10 |
| 1 vs 3 | -2.75 | **0.006** | -0.16 | -0.27 | -0.05 |
| 1 vs 4 | 3.05 | **0.003** | 0.18 | 0.06 | 0.29 |
| 1 vs 5 | -1.98 | **0.049** | -0.12 | -0.23 | 0.00 |
| 1 vs 6 | -6.17 | **0.001*** | -0.36 | -0.48 | -0.24 |
| 2 vs 3 | -2.43 | **0.016** | -0.14 | -0.26 | -0.03 |
| 2 vs 4 | 3.37 | **0.001*** | 0.20 | 0.08 | 0.31 |
| 2 vs 5 | -1.66 | 0.098 | -0.10 | -0.21 | 0.02 |
| 2 vs 6 | -5.85 | **0.001*** | -0.34 | -0.46 | -0.22 |
| 3 vs 4 | 5.69 | **0.001*** | 0.33 | 0.21 | 0.45 |
| 3 vs 5 | 0.83 | 0.405 | 0.05 | -0.07 | 0.16 |
| 3 vs 6 | -3.27 | **0.001** | -0.19 | -0.30 | -0.07 |
| 4 vs 5 | -5.03 | **0.001*** | -0.29 | -0.41 | -0.18 |
| 4 vs 6 | -9.15 | **0.001*** | -0.53 | -0.65 | -0.41 |
| 5 vs 6 | -4.23 | **0.001*** | -0.25 | -0.36 | -0.13 |
| ***Estimated Marginal Means (EMM)*** | | | | | |
| **Experimental Session** | | **EMM (%)** | **SE** | **Lower CI** | **Upper CI** |
| Best Contact, Low Amplitude | | 4.56 | 0.50 | 3.56 | 5.56 |
| Best Contact, Clinical Amplitude | | 4.70 | 0.50 | 3.70 | 5.71 |
| Best Contact, High Amplitude | | 5.81 | 0.51 | 4.78 | 6.83 |
| Worst Contact, Low Amplitude | | 3.23 | 0.50 | 2.23 | 4.23 |
| Worst Contact, Clinical Amplitude | | 5.43 | 0.50 | 4.43 | 6.43 |
| Worst Contact, High Amplitude | | 7.32 | 0.51 | 6.31 | 8.34 |

*Note.* Cohen’s d effect sizes and associated 95% confidence intervals (CI) were calculated based on appropriate test statistics using the *effectsize* package in R. Bolded values indicate statistical significance and trending effects based on *p_corrected_* < .05 and .10, respectively following Tukey’s correction for multiple comparisons and non-overlapping confidence intervals of effect sizes. **p_corrected_* < .001. 1 = best contact, low amplitude; 2 = best contact, clinical amplitude; 3 = best contact, high amplitude; 4 = worst contact, low amplitude; 5 = worst contact, clinical amplitude; 6 = worst contact, high amplitude.

**Supplementary Table 23.** **Model results of experimental session on MFG medium-latency evoked response amplitude during STN-DBS.**

| ***MFG Medium-Latency Evoked Response Amplitude*** | | | | | |
| --- | --- | --- | --- | --- | --- |
| **Effect** | **F** | **p** | **Effect Size (d)** | **Lower CI** | **Upper CI** |
| Experimental Session | 6.96 | **0.001*** | 0.31 | 0.08 | 0.54 |
| ***Post Hoc Testing*** | | | | | |
| **Main Effect of Experimental Session** | **t** | **p** | **Effect Size (d)** | **Lower CI** | **Upper CI** |
| 1 vs 2 | -0.87 | 0.385 | -0.05 | -0.16 | 0.06 |
| 1 vs 3 | -1.81 | 0.072 | -0.11 | -0.22 | 0.01 |
| 1 vs 4 | 0.23 | 0.814 | 0.01 | -0.10 | 0.13 |
| 1 vs 5 | -0.60 | 0.548 | -0.03 | -0.15 | 0.08 |
| 1 vs 6 | -4.86 | **0.001*** | -0.28 | -0.40 | -0.17 |
| 2 vs 3 | -0.95 | 0.343 | -0.06 | -0.17 | 0.06 |
| 2 vs 4 | 1.10 | 0.271 | 0.06 | -0.05 | 0.18 |
| 2 vs 5 | 0.27 | 0.789 | 0.02 | -0.10 | 0.13 |
| 2 vs 6 | -4.01 | **0.001*** | -0.23 | -0.35 | -0.12 |
| 3 vs 4 | 2.04 | **0.043** | 0.12 | 0.00 | 0.23 |
| 3 vs 5 | 1.21 | 0.226 | 0.07 | -0.04 | 0.18 |
| 3 vs 6 | -3.01 | **0.003** | -0.17 | -0.29 | -0.06 |
| 4 vs 5 | -0.84 | 0.404 | -0.05 | -0.16 | 0.07 |
| 4 vs 6 | -5.09 | **0.001*** | -0.30 | -0.41 | -0.18 |
| 5 vs 6 | -4.27 | **0.001*** | -0.25 | -0.36 | -0.13 |
| ***Estimated Marginal Means (EMM)*** | | | | | |
| **Experimental Session** | | **EMM (%)** | **SE** | **Lower CI** | **Upper CI** |
| Best Contact, Low Amplitude | | 4.66 | 0.47 | 3.71 | 5.62 |
| Best Contact, Clinical Amplitude | | 5.03 | 0.47 | 4.08 | 5.98 |
| Best Contact, High Amplitude | | 5.44 | 0.48 | 4.47 | 6.41 |
| Worst Contact, Low Amplitude | | 4.56 | 0.47 | 3.61 | 5.52 |
| Worst Contact, Clinical Amplitude | | 4.92 | 0.47 | 3.96 | 5.87 |
| Worst Contact, High Amplitude | | 6.75 | 0.48 | 5.79 | 7.72 |

*Note.* Cohen’s d effect sizes and associated 95% confidence intervals (CI) were calculated based on appropriate test statistics using the *effectsize* package in R. Bolded values indicate statistical significance and trending effects based on *p_corrected_* < .05 and .10, respectively following Tukey’s correction for multiple comparisons and non-overlapping confidence intervals of effect sizes. **p_corrected_* < .001. 1 = best contact, low amplitude; 2 = best contact, clinical amplitude; 3 = best contact, high amplitude; 4 = worst contact, low amplitude; 5 = worst contact, clinical amplitude; 6 = worst contact, high amplitude.

**Supplementary Table 24.** **Model results of experimental session on SMA medium-latency evoked response amplitude during STN-DBS.**

| ***SMA Medium-Latency Evoked Response Amplitude*** | | | | | |
| --- | --- | --- | --- | --- | --- |
| **Effect** | **F** | **p** | **Effect Size (d)** | **Lower CI** | **Upper CI** |
| Experimental Session | 5.53 | **0.001*** | 0.27 | 0.04 | 0.50 |
| ***Post Hoc Testing*** | | | | | |
| **Main Effect of Experimental Session** | **t** | **p** | **Effect Size (d)** | **Lower CI** | **Upper CI** |
| 1 vs 2 | -1.42 | 0.158 | -0.08 | -0.19 | 0.03 |
| 1 vs 3 | -2.03 | **0.043** | -0.12 | -0.23 | 0.00 |
| 1 vs 4 | -1.78 | 0.076 | -0.10 | -0.22 | 0.01 |
| 1 vs 5 | -1.16 | 0.245 | -0.07 | -0.18 | 0.05 |
| 1 vs 6 | -4.97 | **0.001*** | -0.29 | -0.40 | -0.17 |
| 2 vs 3 | -0.63 | 0.523 | -0.04 | -0.15 | 0.08 |
| 2 vs 4 | -0.36 | 0.716 | -0.02 | -0.13 | 0.09 |
| 2 vs 5 | 0.25 | 0.802 | 0.01 | -0.10 | 0.13 |
| 2 vs 6 | -3.55 | **0.001*** | -0.20 | -0.32 | -0.09 |
| 3 vs 4 | 0.28 | 0.779 | 0.02 | -0.10 | 0.13 |
| 3 vs 5 | 0.89 | 0.376 | 0.05 | -0.06 | 0.16 |
| 3 vs 6 | -2.85 | **0.005** | -0.16 | -0.28 | -0.05 |
| 4 vs 5 | 0.61 | 0.539 | 0.04 | -0.08 | 0.15 |
| 4 vs 6 | -3.19 | **0.002** | -0.18 | -0.30 | -0.07 |
| 5 vs 6 | -3.80 | **0.001*** | -0.22 | -0.33 | -0.10 |
| ***Estimated Marginal Means (EMM)*** | | | | | |
| **Experimental Session** | | **EMM (%)** | **SE** | **Lower CI** | **Upper CI** |
| Best Contact, Low Amplitude | | 3.99 | 0.43 | 3.13 | 4.85 |
| Best Contact, Clinical Amplitude | | 4.69 | 0.43 | 3.83 | 5.54 |
| Best Contact, High Amplitude | | 5.01 | 0.44 | 4.13 | 5.88 |
| Worst Contact, Low Amplitude | | 4.87 | 0.43 | 4.01 | 5.72 |
| Worst Contact, Clinical Amplitude | | 4.56 | 0.43 | 3.71 | 5.42 |
| Worst Contact, High Amplitude | | 6.43 | 0.43 | 5.57 | 7.29 |

*Note.* Cohen’s d effect sizes and associated 95% confidence intervals (CI) were calculated based on appropriate test statistics using the *effectsize* package in R. Bolded values indicate statistical significance and trending effects based on *p_corrected_* < .05 and .10, respectively following Tukey’s correction for multiple comparisons and non-overlapping confidence intervals of effect sizes. **p_corrected_* < .001. 1 = best contact, low amplitude; 2 = best contact, clinical amplitude; 3 = best contact, high amplitude; 4 = worst contact, low amplitude; 5 = worst contact, clinical amplitude; 6 = worst contact, high amplitude.

**Supplementary Table 25. Achieved loadings derived from principal components analyses of finger tapping dynamics.**

| ***Finger Tapping Movement Profiles*** | | | |
| --- | --- | --- | --- |
| **Metric** | **Achieved**  **Loadings** | **Eigenvalue** | **Variance**  **Accounted For** |
| Acceleration Magnitude | 0.93 | 3.50 | 70.0% |
| Acceleration Variability | 0.73 |  |  |
| Inter-tap Interval | 0.84 |  |  |
| Acceleration Jerk | 0.89 |  |  |
| Jerk Variability | 0.79 |  |  |

*Note*. Kinematic features of finger tapping performance were subjected to principal components analyses to derive a single component of finger-tapping movement profiles. Reverse coded acceleration magnitude, reverse coded acceleration jerk (i.e., movement smoothness), inter-tap interval and the coefficient of variation in acceleration magnitude and jerk accounted for 70.0% of the variance in finger-tapping movement profiles.

**Supplementary Table 26. Model results of mediation analysis using Baron and Kenny (1986) regression method of finger tapping movement profiles on SM1 HDP-related activation and beta oscillatory power.**

| ***Mediation Analysis*** | | | | | |
| --- | --- | --- | --- | --- | --- |
| **Simple regression of SM1 beta oscillations on HDP-related activation** | **t** | **p** | **Effect Size (d)** | **Lower CI** | **Upper CI** |
| HDP-related activation | 3.99 | **0.001*** | 0.81 | 0.39 | 1.22 |
| Experimental Session | 2.47 | **0.016** | 0.50 | 0.10 | 0.90 |
| Interaction | -2.56 | **0.012** | -0.52 | -0.92 | -0.11 |
| **Simple regression of finger tapping performance on SM1 HDP-related activation** | **t** | **p** | **Effect Size (d)** | **Lower CI** | **Upper CI** |
| HDP-related activation | -2.33 | **0.022** | -0.49 | -0.91 | -0.07 |
| Experimental Session | -2.21 | **0.03** | -0.50 | -0.94 | -0.05 |
| Interaction | 2.93 | **0.004** | 0.65 | 0.20 | 1.09 |
| **Multiple regression of finger tapping performance on SM1 beta oscillations and HDP-related activation** | **t** | **p** | **Effect Size (d)** | **Lower CI** | **Upper CI** |
| HDP-related activation | -0.10 | 0.918 | -0.02 | -0.43 | 0.39 |
| Experimental Session | 1.26 | 0.212 | 0.26 | -0.15 | 0.68 |
| Beta Power | -1.04 | 0.303 | -0.22 | -0.63 | 0.19 |

*Note.* Cohen’s d effect sizes and associated 95% confidence intervals (CI) were calculated based on appropriate test statistics using the *effectsize* package in R. Bolded values indicate statistical significance and trending effects based on *p_corrected_* < .05 and .10, respectively following Tukey’s correction for multiple comparisons and non-overlapping confidence intervals of effect sizes. **p_corrected_* < .001.


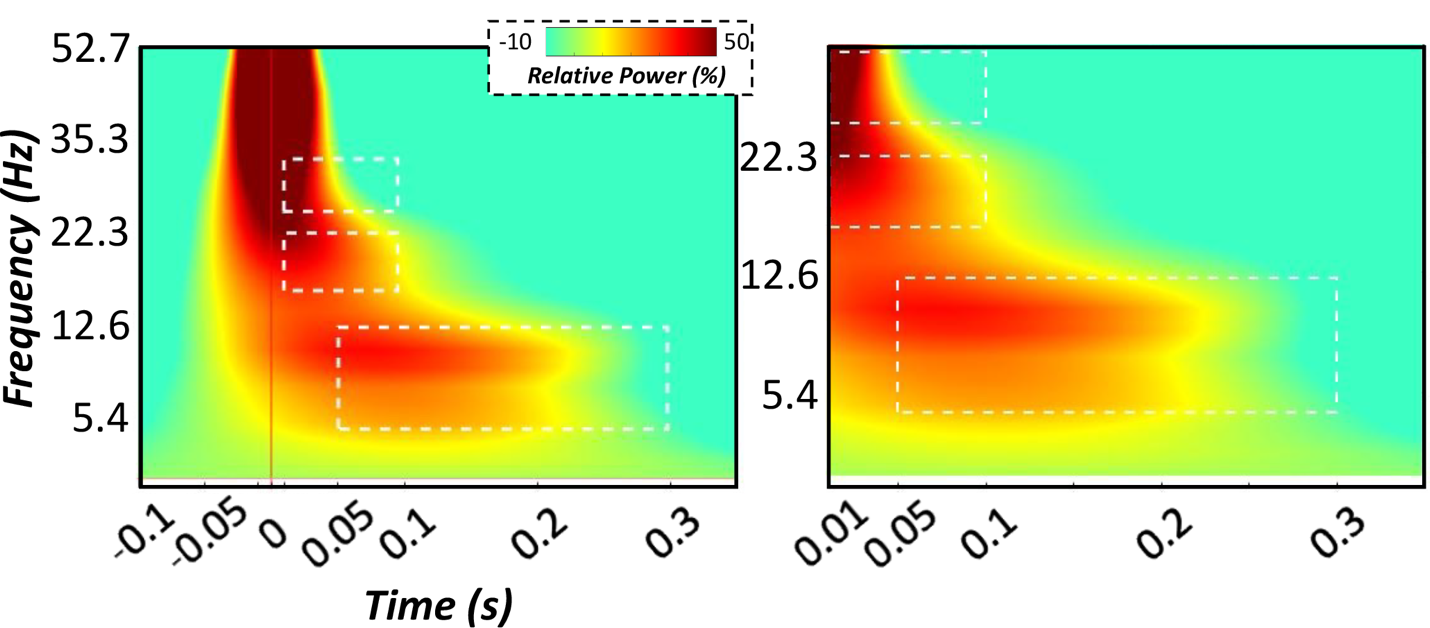


**Supplementary Figure 1. Significant Time-Frequency Windows Identified at the Sensor Level for Source Imaging Analyses.** (Left and Right panel) Grand-averaged time-frequency spectrogram (i.e., averaged across all six experimental conditions: best and worst contacts, low, clinical and high stimulation amplitudes) over the sensorimotor cortex ipsilateral to the site of STN stimulation (MEG0432). Time-point zero denotes DBS pulse onset. The baseline window was defined as the -100 to -5 ms window prior to stimulation pulse onset. Power is shown as percentage units relative to the baseline period, with the color scale bar fixed for left and right panels. White dashed boxes denote significant time and frequency windows identified for theta-alpha (i.e., 4-12 Hz from 50-300 ms), low beta (i.e., 14-22 Hz from 10-100 ms) and high beta (i.e., 24-30 Hz from 10-100 ms) oscillatory responses and were subjected to source reconstruction using weighted minimum norm estimation. Of note, oscillatory response time windows were shifted by at least 10-50 ms surrounding response maxima following DBS pulse onset to avoid remnants of the DBS artifact and to optimize the signal to noise ratio surrounding response maxima (i.e., greatest amplitude change from baseline). For a more focused view of the aforementioned time-frequency segments, see the right panel starting 10 ms following DBS pulse onset.


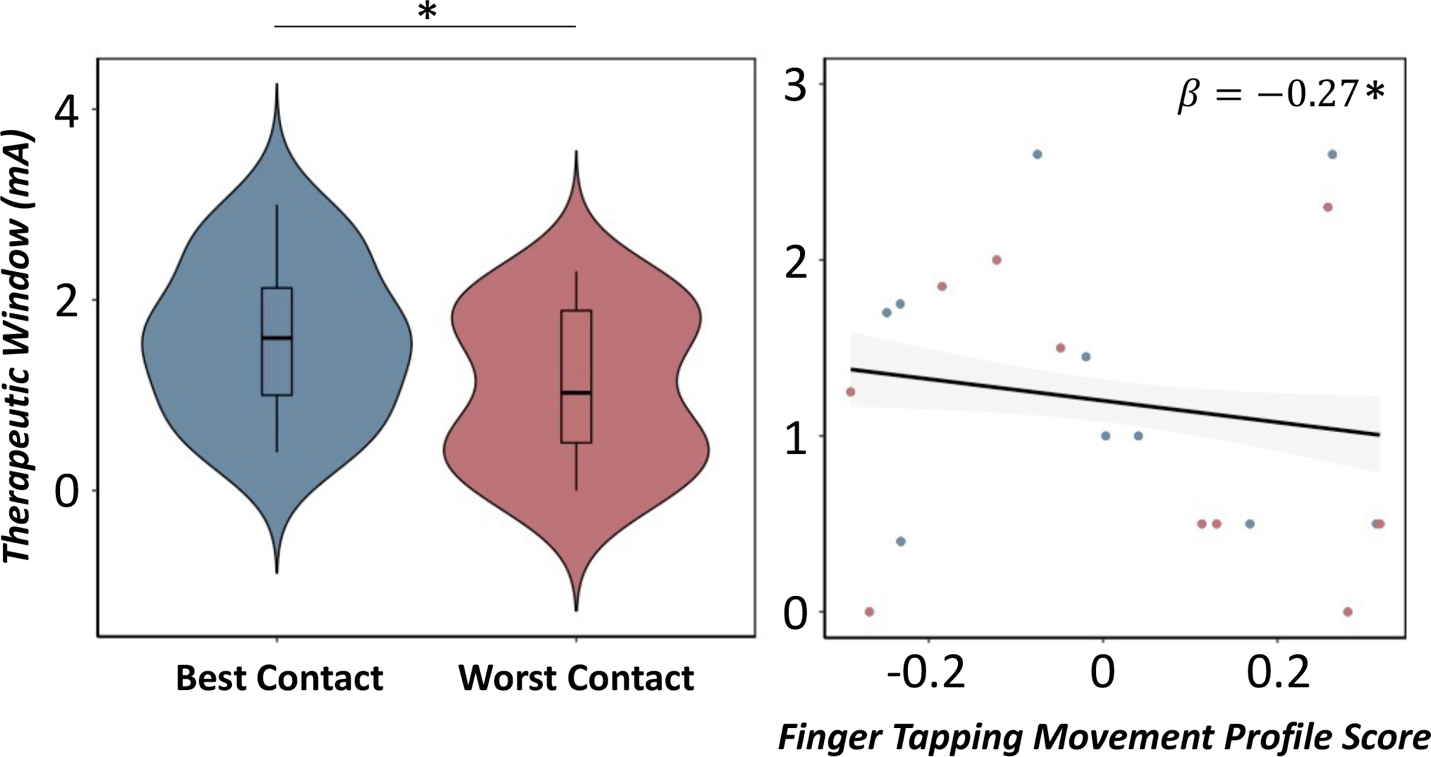


**Supplementary Figure 2. Quantitative Finger Tapping Movement Profiles Relate to Traditional Clinical Outcomes.** Linear mixed effects models of clinically-effect contact and amplitude settings (session factor with 2 levels; left panel) on therapeutic windows were conducted. As expected, therapeutic windows were significantly modulated by DBS contact settings, with greater therapeutic windows (i.e., minimal distance in mA from observed clinical effect to observed side effect) achieved during clinically-effective “best” contact settings compared to worse contact settings. Next, we conducted a linear mixed effect model of finger tapping movement profiles on therapeutic windows was conducted with subject and DBS contact setting included as a nested random effect. Lower movement profile scores (i.e., better behavioral performance) were predictive of greater therapeutic windows (i.e., better clinical outcome), regardless of DBS contact setting. Taken together, these data suggest that quantitatively-derived finger tapping movement profiles as measured in the current study relate to traditional clinical evaluations of appropriate DBS parameter settings. 95% confidence intervals are displayed in gray for the regression line. LME: **p* < .05.
