# Supplementary material for: Modulation of DBS-induced cortical responses and movement by the directionality and magnitude of current administered
Source: NPJ Parkinsons Dis. 2024 Mar 8;10:53. doi: 10.1038/s41531-024-00663-9 (PMC10923868; doi:10.1038/s41531-024-00663-9)
Supplement: Supplementary file 2 — Reporting Summary [file 41531_2024_663_MOESM2_ESM.pdf]

nature portfolio

Corresponding author(s): Rachel Spooner and Esther Florin

Last updated by author(s): Jan 31, 2024

Reporting Summary

Nature Portfolio wishes to improve the reproducibility of the work that we publish. This form provides structure for consistency and transparency in reporting. For further information on Nature Portfolio policies, see our Editorial Policies and the Editorial Policy Checklist.

Statistics

For all statistical analyses, confirm that the following items are present in the figure legend, table legend, main text, or Methods section.

n/a	Confirmed	
☐	☒	The exact sample size (*n*) for each experimental group/condition, given as a discrete number and unit of measurement
☐	☒	A statement on whether measurements were taken from distinct samples or whether the same sample was measured repeatedly
☐	☒	The statistical test(s) used AND whether they are one- or two-sided<br>*Only common tests should be described solely by name; describe more complex techniques in the Methods section.*
☐	☒	A description of all covariates tested
☐	☒	A description of any assumptions or corrections, such as tests of normality and adjustment for multiple comparisons
☐	☒	A full description of the statistical parameters including central tendency (e.g. means) or other basic estimates (e.g. regression coefficient) AND variation (e.g. standard deviation) or associated estimates of uncertainty (e.g. confidence intervals)
☐	☒	For null hypothesis testing, the test statistic (e.g. *F*, *t*, *r*) with confidence intervals, effect sizes, degrees of freedom and *P* value noted<br>*Give P values as exact values whenever suitable.*
☒	☐	For Bayesian analysis, information on the choice of priors and Markov chain Monte Carlo settings
☐	☒	For hierarchical and complex designs, identification of the appropriate level for tests and full reporting of outcomes
☐	☒	Estimates of effect sizes (e.g. Cohen's *d*, Pearson's *r*), indicating how they were calculated

Our web collection on statistics for biologists contains articles on many of the points above.

Software and code

Policy information about availability of computer code

Data collection	The St. Jude Medical Infinity DBS System Clinical Programmer was used to test DBS parameter settings during MEG and behavioral recordings.
Data analysis	Our MEG and accelerometer data analysis pipelines were conducted using open source software (i.e., Brainstorm (Tadel et al., 2011) and custom algorithms in Matlab (Version 2021a). All statistical analyses were conducted using R (Version 4.0.3).

For manuscripts utilizing custom algorithms or software that are central to the research but not yet described in published literature, software must be made available to editors and reviewers. We strongly encourage code deposition in a community repository (e.g. GitHub). See the Nature Portfolio guidelines for submitting code & software for further information.

Data

Policy information about availability of data

All manuscripts must include a data availability statement. This statement should provide the following information, where applicable:

- Accession codes, unique identifiers, or web links for publicly available datasets
- A description of any restrictions on data availability
- For clinical datasets or third party data, please ensure that the statement adheres to our policy

The anonymized data from this study will be made available to investigators upon request from the corresponding authors.

Research involving human participants, their data, or biological material

Policy information about studies with human participants or human data. See also policy information about sex, gender (identity/presentation), and sexual orientation and race, ethnicity and racism.

Reporting on sex and gender	Sex- and gender-based analyses were omitted from the current study as our cohort was comprised of approximately 90% males, making it an unbalanced cohort for formal analysis. Importantly, this sex and gender distribution is common in other studies of Parkinson's disease, as 1.5-2 times more men are afflicted by the disease than women globally. Thus, our sample corresponds well to this male-dominant disease prevalence.
Reporting on race, ethnicity, or other socially relevant groupings	Reporting on race, ethnicity or other socially relevant groupings was not included in the current study as it was outside the scope and our sample size was too small to power such analyses.
Population characteristics	Population characteristics were not included in the formal analyses as they were outside the scope of the current study.
Recruitment	Twenty patients with Parkinson’s disease (Mage = 63.96 years old, 43-80 years old, 3 females) implanted with STN-DBS (Abbott Infinity DBS System, lead model: 6172, electrode: 6671, Abbott, Plano, Texas, USA) were recruited for this study from the Center for Movement Disorders and Neuromodulation at the University Hospital Düsseldorf. Exclusionary criteria included any medical illness affecting CNS function, any neurological or psychiatric disorder (except PD), severe depression (Beck Depression Inventory > 30), or cognitive impairment (Mini-Mental State Examination < 26).
Ethics oversight	The local ethics committee at the University Hospital Duesseldorf approved the study (No. 2019-626_2) and all patients provided written informed consent in accordance with the Declaration of Helsinki.

Note that full information on the approval of the study protocol must also be provided in the manuscript.

Field-specific reporting

Please select the one below that is the best fit for your research. If you are not sure, read the appropriate sections before making your selection.

☒ Life sciences ☐ Behavioural & social sciences ☐ Ecological, evolutionary & environmental sciences

For a reference copy of the document with all sections, see nature.com/documents/nr-reporting-summary-flat.pdf

Life sciences study design

All studies must disclose on these points even when the disclosure is negative.

Sample size	Based on previous studies from our laboratory and others evaluating the neurophysiological correlates of DBS therapy in PD patients, concomitant with effect size and confidence interval calculations based on relevant test statistics for each reported result (see Supplementary Materials), we derived that 20 participants would provide adequate power for all behavioral and neural analyses. Power analyses were conducted using the simr package in R (Version 4.0.3) using 1000 simulations and a seed of 4533. Effect size and associated confidence intervals were computed based on the appropriate test statistics using the effectsize package in R (Version 4.0.3).
Data exclusions	Of the 20 patients enrolled in the current study, 2 patients were unable to successfully complete the MEG and behavioral aspects of the study.
Replication	Effect size (Cohen's d and r) and associated confidence interval estimations for each trending and significant main effect reported in the current study were computed using the effectsize package in R (Version 4.0.3).
Randomization	The administration of alternating DBS parameter settings (i.e., contact and stimulation amplitudes) during MEG and behavioral data acquisition were pseudorandomized.
Blinding	The administration of varying DBS parameter settings (i.e., contact and stimulation amplitude settings) were not blinded to the investigator responsible for data acquisition in the current study.

Reporting for specific materials, systems and methods

We require information from authors about some types of materials, experimental systems and methods used in many studies. Here, indicate whether each material, system or method listed is relevant to your study. If you are not sure if a list item applies to your research, read the appropriate section before selecting a response.

Materials & experimental systems

n/a	Involved in the study
☒	☐ Antibodies
☒	☐ Eukaryotic cell lines
☒	☐ Palaeontology and archaeology
☒	☐ Animals and other organisms
☒	☐ Clinical data
☒	☐ Dual use research of concern
☒	☐ Plants

Methods

n/a	Involved in the study
☒	☐ ChIP-seq
☒	☐ Flow cytometry
☐	☒ MRI-based neuroimaging

Plants

Seed stocks	Not applicable.
Novel plant genotypes	Not applicable.
Authentication	Not applicable.

Magnetic resonance imaging

Experimental design

Design type	Pre-surgical T1-weighted structural MRIs were acquired for patients included in the current study.
Design specifications	This study only included structural MRI data for coregistration to functional MEG data in our analysis pipeline.
Behavioral performance measures	No behavioral measures were acquired during MRI sessions.

Acquisition

Imaging type(s)	Structural
Field strength	3
Sequence & imaging parameters	T1-weighted MPRAGE
Area of acquisition	A whole-brain acquisition was completed.

Diffusion MRI ☐ Used ☒ Not used

Preprocessing

Preprocessing software	Structural MRI segmentation was conducted using the CAT12 Toolbox in SPM12.
Normalization	MEG source level data (i.e., weighted minimum norm estimate 3D maps of source power) were normalized to the baseline period in the experimental epoch to identify significant neural activation patterns. This normalization step was conducted using the open-source software, Brainstorm (Tadel et al., 2011).
Normalization template	Source reconstructed MEG data were normalized and projected to MNI space (ICBM125 2009c Nonlinear Asymmetric) for group-level analyses.
Noise and artifact removal	MEG data were subjected to noise reduction using the signal-space separation method with a temporal extension (tSSS; Taulu et al., 2006). Cardiac and ocular artifacts in the MEG data were removed from the data using signal space projection (SSP; Uusitalo et al., 1997).
Volume censoring	*Define your software and/or method and criteria for volume censoring, and state the extent of such censoring.*

Statistical modeling & inference

Model type and settings

Linear mixed effects models of neural and behavioral parameters on experimental session (fixed effect factor with 6 levels), controlling for subject as a random effect were conducted using the lme4 package in R (Version 4.0.3).

Effect(s) tested

The effect of experimental session (factor with 6 levels), neural response amplitude (continuous) and their interaction were included as fixed effects in our models of brain-behavior relationships.

Specify type of analysis: ☐ Whole brain ☒ ROI-based ☐ Both

Anatomical location(s)

Significant deviations in neural evoked response amplitude and induced oscillatory response amplitude from baseline were identified in the sensor-level MEG data by first conducting paired-sample t-tests against baseline across subjects, followed up with non-parametric permutation testing to control for multiple comparisons (initial threshold: $p < .05$, permutations: 10,000). The permutation procedure used Monte Carlo random sampling to estimate the empirical distribution of the t-statistic at each sensor, time point and frequency in the experimental epoch where appropriate. The resulting phase-locked, time-domain period and time-frequency window that significantly differed from baseline (FDR-corrected at $p < .005$ and minimum duration of 5 ms for time and sensors) was used to guide subsequent time-domain source-level analyses to select the time window of interest. Using the temporal and spectral clusters identified in the sensor-level analysis, source-level MEG data were grand-averaged using the dominant orientation (i.e., greatest amplitude increase/decrease from baseline) over the significant time and/or frequency windows identified at the sensor level across all trials, experimental runs and patients to determine the peak vertex (i.e., ROI) of the time-domain neural response.

Statistic type for inference

(See Eklund et al. 2016)

Grand-averaged source level data were displayed at an amplitude threshold of 30% with a minimum vertex-wise cluster of 20 vertices to determine the peak vertex (i.e., ROI) of the time-domain and oscillatory neural responses as a function of DBS pulses.

Correction

Statistical analysis of the sensor level MEG data were corrected for multiple comparisons using FDR-corrected non-parametric permutation testing using Monte Carlo random sampling ($p < .005$, minimum duration of 5 ms for time and sensors).

Models & analysis

n/a	Involved in the study
☒	☐ Functional and/or effective connectivity
☒	☐ Graph analysis
☐	☒ Multivariate modeling or predictive analysis

Multivariate modeling and predictive analysis

Linear mixed effects models (LMEs) and mediation analyses of experimental session (i.e., best and worst contacts tested at clinically effective stimulation amplitudes +/-50%: fixed effect factor with 6 levels) controlling for subject (random effect) on behavioral and neural outcomes were conducted separately using the lme4 package in R (Version 4.0.3). All LME post-hoc analyses of trending and significant main effects were corrected for multiple comparisons using Tukey's multiple comparison test. Relevant behavioral metrics were subjected to an exploratory factor analysis and we progressively removed individual variables based on poor loadings ($\lambda < .70$), and overall model fit. Criteria for good model fit included a non-statistically significant chi square, a root mean squared error approximation (RMSEA) < .06, a comparative fit index (CFI) > .95, and a standardized root mean squared residual (SRMR) < .08 based on standards in the literature. The best fitting model was used to define a latent variable for which a movement profile score was extracted per participant. Modeling and component extraction was completed using lavaan and principal functions in R (Version 4.0.3), respectively.
